# Low‐Energy‐Consumption and Electret‐Free Photosynaptic Transistor Utilizing Poly(3‐hexylthiophene)‐Based Conjugated Block Copolymers

**DOI:** 10.1002/advs.202105190

**Published:** 2022-01-22

**Authors:** Wei‐Chen Yang, Yan‐Cheng Lin, Shin Inagaki, Hiroya Shimizu, Ender Ercan, Li‐Che Hsu, Chu‐Chen Chueh, Tomoya Higashihara, Wen‐Chang Chen

**Affiliations:** ^1^ Department of Chemical Engineering National Taiwan University Taipei 10617 Taiwan; ^2^ Advanced Research Center for Green Materials Science and Technology National Taiwan University Taipei 10617 Taiwan; ^3^ Department of Organic Materials Science Graduate School of Organic Materials Science Yamagata University Yonezawa Yamagata 992‐8510 Japan; ^4^ Institute of Polymer Science and Engineering National Taiwan University Taipei 10617 Taiwan

**Keywords:** field‐effect transistor, pair‐pulse facilitation, photonic synapses, poly(3‐hexylthiophene), ultrafast photoresponse

## Abstract

Neuromorphic computation possesses the advantages of self‐learning, highly parallel computation, and low energy consumption, and is of great promise to overcome the bottleneck of von Neumann computation. In this work, a series of poly(3‐hexylthiophene) (P3HT)‐based block copolymers (BCPs) with different coil segments, including polystyrene, poly(2‐vinylpyridine) (P2VP), poly(2‐vinylnaphthalene), and poly(butyl acrylate), are utilized in photosynaptic transistor to emulate paired‐pulse facilitation, spike time/rate‐dependent plasticity, short/long‐term neuroplasticity, and learning−forgetting−relearning processes. P3HT serves as a carrier transport channel and a photogate, while the insulating coils with electrophilic groups are for charge trapping and preservation. Three main factors are unveiled to govern the properties of these P3HT‐based BCPs: i) rigidity of the insulating coil, ii) energy levels between the constituent polymers, and iii) electrophilicity of the insulating coil. Accordingly, P3HT‐*b*‐P2VP‐based photosynaptic transistor with a sought‐after BCP combination demonstrates long‐term memory behavior with current contrast up to 10^5^, short‐term memory behavior with high paired‐pulse facilitation ratio of 1.38, and an ultralow energy consumption of 0.56 fJ at an operating voltage of −0.0003 V. As far as it is known, this is the first work to utilize conjugated BCPs in an electret‐free photosynaptic transistor showing great potential to the artificial intelligence technology.

## Graphical abstract

A series of poly(3‐hexylthiophene) (P3HT)‐based block copolymers with different coil segments, including polystyrene, poly(2‐vinylpyridine) (P2VP), poly(2‐vinylnaphthalene), and poly(butyl acrylate), are utilized in photosynaptic transistor to emulate pair‐pulse facilitation, spike time/rate‐dependent plasticity, short/long‐term neuroplasticity, and learning−forgetting−relearning processes. Accordingly, P3HT‐*b*‐P2VP‐based device shows an ultralow energy consumption of 0.56 fJ at an operating voltage of −0.0003 V.

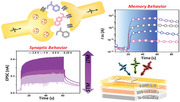

## 1 Introduction

With the explosive growth of information, the von Neumann traditional computing method constructed by five major subsystems (memory, arithmetic logic unit, control unit, input, and output) gradually faces the challenges of high energy consumption of data processing, low computing speed, and low data capacity.^[^ ^1^, ^2^ ^]^ Therefore, using an electronic device with photocommunication capability must become the mainstream technology in the future. It is understood that light can play multifunctions in a photosensitive device including: i) as a stimulus of the input signal, ii) as an auxiliary adjustment method for electrical stimulation, and iii) as a response to the output signal. Collectively, noncontact programming with a light stimulus grants the advantages of extensive operations with broad light bandwidth, low crosstalk, energy consumption, and RC (resistive–capacitive) delay.^[^ ^3^, ^4^ ^]^ Recently, phototransistors mimicking the human visual system with the functions of sensing, recognition, reception, and memorizing the environmental information have received huge research attention among the various photosensitive devices. Notably, phototransistors are extensively applied in nonvolatile memory which is a type of computer memory that can retain the stored information even after removing the power. In addition, phototransistors are also applicable in artificial synapse to fulfill short/long‐term memory or plasticity and multilevel information recording.

With regard to the long‐term memory application, phototransistor memory is accordingly developed. In the basic operation of a nonvolatile flash memory, the photogenerated excitons in the semiconducting channel or floating gate electret separate into free charges, and parts of these charges subsequently tunnel into the channel while the countercharges are trapped in the electret. Therefore, the insulating medium and the photogates play an important role in the charge retention of a memory device, and many designs on floating gate electrets are vigorously developed.^[^ ^5^, ^6^, ^7^ ^]^ The purpose of these approaches shares similar concepts of forming discrete photogates in an insulating medium to promote the devices performance, and an insulating medium with a suitable energy level and/or certain functionality stabilizes the charges to exhibit an admirable capability of hole or electron trapping. However, polymer blends or hybrid composites are bound to be limited in the future development because of the morphological inhomogeneity with the aggregation of photogates, and the solvent selectivity among the constituent materials is another obstacle.^[^ ^8^ ^]^ In addition, integration of the semiconducting channel and the electret such as forming hybrid composite will compromise the carrier transport and charge trapping of a memory device.^[^ ^9^, ^10^, ^11^, ^12^, ^13^ ^]^ Therefore, it would be of great significance to develop photogating materials with both carrier transport and charge storage characteristics for phototransistors.

With regard to the short‐term memory and artificial synapse applications, the “neuromorphic computing” proposed in the late 1980s by Carver Mead, IBM's TrueNorth chip and Intel's Loihi chip evidenced neuromorphic circuits and synaptic functionalities based on a silicon complementary metal–oxide–semiconductor (CMOS), and the first synaptic device consisting of nonvolatile memristors was demonstrated by HP Labs in 2008. Later on, phase‐change, atomic‐switched, and ferroelectric memory devices have been diversely developed as new NVMs to mimic synaptic functionalities. Photonic synaptic devices may enable the bidirectional conversion between electrical and optical signals, and this property can significantly facilitate the integration of optoelectronic neuromorphic computing systems.^[^ ^14^ ^]^ With regard to the merits earlier discussed, photosynaptic transistors have gained increasing importance in photocommunication technology, which play a pivotal role in biological perception and synaptic computation systems.^[^ ^15^, ^16^ ^]^

The human visual system can recognize nearly 80% of information from the complex external environment, and therefore, the development of photosynaptic components can be the foundation for human visual simulation with the brain comprising ≈10^11^ neurons and ≈10^15^ synapses. Neurons are the basic unit of human brain function, and synapses are important building blocks for neuron signal transmission and exchange.^[^ ^17^, ^18^ ^]^ The level of postsynaptic current is determined by the synaptic weight defined as the strength of communication between the pre/postneurons, and the postsynaptic current is relating to charge trapping/detrapping in a photosynaptic transistor.^[^ ^19^, ^20^ ^]^ To date, the reported photosynaptic transistors have successfully emulated the fundamental synaptic functions, such as short‐term plasticity (STP), paired‐pulse facilitation (PPF), long‐term plasticity (LTP), and the short‐term memory (STM) to long‐term memory (LTM) transitions by light stimuli.^[^ ^21^, ^22^ ^]^ Various kinds of materials have been employed for photosynaptic devices, such as metal oxides, perovskites, and organic materials, Kumar et al. reported highly transparent photonic synapses based on fluorine‐doped ZnO. The photogenerated carriers in ZnO conferred by UV light were separated at the interface and captured by the potential walls to simulate the STP, PPF, and LTP synaptic behaviors.^[^ ^23^ ^]^ Zhu. et al. studied a MAPbI_3_‐based device with a synaptic function by regulating the intensity or light wavelength to control the iodine vacancy generation and annihilation that present memory formation and loss behaviors.^[^ ^24^ ^]^ Dai et al. introduced a polyacrylontrile dielectric layer in a C_8_‐BTBT organic transistor. The photoinduced carriers were trapped by a large number of polymer polar groups to mimic synaptic functions. In addition, the dynamic learning and forgetting processes were simulated through transistor arrays without using a silicon substrate.^[^ ^25^ ^]^ However, the morphology of floating gate electret and its integration to the semiconducting channel will largely influence the synaptic behaviors of a photosynaptic transistor.

Conjugated molecules or polymers have the advantages of broad optical absorption and tunable carrier transport, but they usually rely on another photogate or floating gate electret to achieve synaptic behavior. Perovskite materials show high photoresponse owing to their wide optical absorption and photoactivity; however, the toxic heavy metal atoms raise the concern in leakage and environmental pollution. In addition, composite materials consisting of conjugated polymers and perovskite quantum dots (QDs) show poor miscibility and dispersity deteriorating the device performance. Therefore, it is trendy to develop high‐performance channel materials for photosynaptic transistor with high homogeneity, simplified fabrication, and environmental friendliness. Conjugated block copolymer (BCP) design holds great promise in concomitantly achieving carrier transport and countercharge trapping.^[^ ^26^ ^]^ However, there is no related report to the design of conjugated BCP for photosynaptic transistor, and there is no systematic study on the effect of self‐assembled microstructure on the device performance. Therefore, it is of great interest to exploit an electret‐free phototransistor device by using conjugated BCPs for both carrier transport and charge trapping to mitigate the issues of morphological inhomogeneity and difficulties in device integration.

In this work, a series of poly(3‐hexylthiophene) (P3HT)‐based BCPs incorporating different coil segments of polystyrene (PS), poly(2‐vinylpyridine) (P2VP), poly(2‐vinylnaphthalene) (PVN), and poly(butyl acrylate) (PBA) were used to fabricate phototransistor. P3HT serves as the carrier transport channel and a photogate, while the insulating segment with an electrophilic group is for countercharge trapping. Morphological analyses by atomic force microscopy (AFM), grazing incident wide‐angle X‐ray scattering (GIWAXS), and time‐resolved photoluminescence (TR‐PL) analyses revealed that BCP films with cluster morphology, high crystallinity, and favorable energy level alignment between the conjugated and insulating segments were conducive to charge storage without sacrificing their carrier transport properties in the derived phototransistor devices. Therefore, electret‐free phototransistors based on P3HT‐BCPs showed good reproducibility, reversibility, and excellent long‐term memory stability without significant dissipation. Furthermore, the phototransistors were capable of emulating synaptic behaviors, including the PPF, spike‐dependent plasticity and STM/LTM behaviors. To the best of our knowledge, this is the first design on low‐energy‐consumption and electret‐free photosynaptic transistor conferred by conjugated BCPs showing great potential for the artificial intelligence technology.

## 2 Results and Discussion

### 2.1 Characterization of P3HT‐Based BCPs

**Figure 1:**
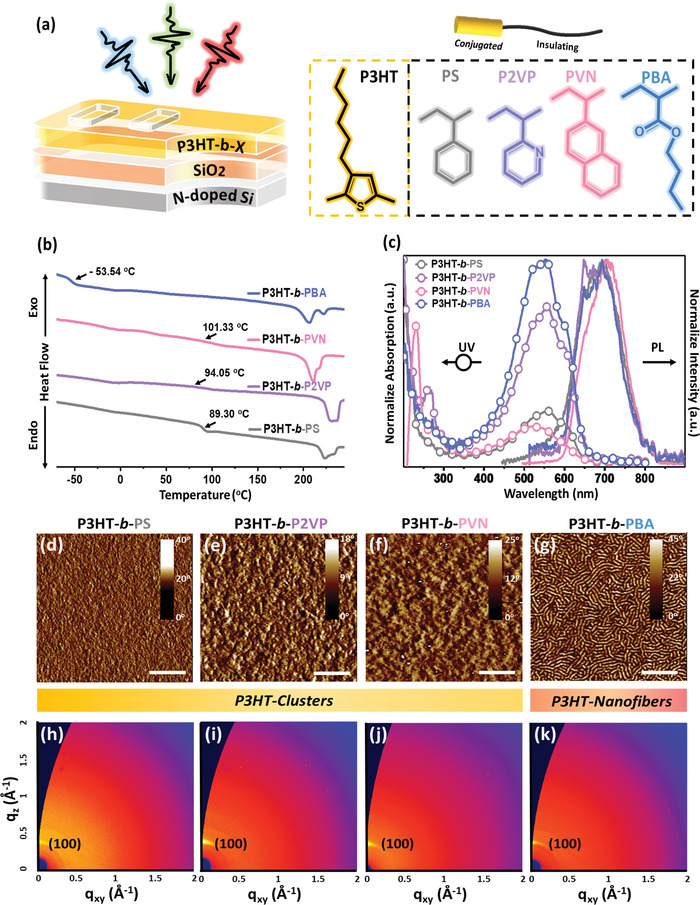
a) Device structure of the photosynaptic transistors and chemical structures of the P3HT‐based BCPs in this study. b) DSC traces of the second heating process and c) UV–vis absorption and PL emission spectra of the of the P3HT‐based BCPs. d–g) AFM phase images with inset scale bar of 2.8 µm and h–k) 2D GIXD profiles of the thermally annealed BCP films of d,h) P3HT‐*b*‐PS, e,i) P3HT‐*b*‐P2VP, f,j) P3HT‐*b*‐PVN, and g,k) P3HT‐*b*‐PBA.

A series of BCPs with varied functional coils, such as PS, P2VP, PVN, and PBA, were synthesized, and their thermal, optical, electrochemical, and crystallographic properties were investigated to understand their structure−property relationship. The chemical structures of the studied BCPs and the bottom‐gate/top‐contact photonic transistor device are displayed in **Figure** **1**a, and the structure characteristics are summarized in Figures S1 and S2 (Supporting Information). The thermal properties, including thermogravimetric (TGA) and differential scanning calorimetry (DSC) analyses, of the BCPs were first investigated and are presented in Figure S3 (Supporting Information) and Figure 1b. As can be seen, these P3HT‐based BCPs possessed typical thermal decomposition temperatures of >300 °C. Based on the DSC profiles, the glass transition temperatures (*T*_g_) for P3HT‐*b*‐PS, P3HT‐*b*‐P2VP, P3HT‐*b*‐PVN, and P3HT‐*b*‐PBA were 89, 94, 101, and −54 °C, respectively, which is attributed to the insulating segments of BCPs, and their melting temperatures were approximately 205−235 °C, which is related to the bimodal melting peaks of the P3HT block.^[^ ^27^ ^]^ Notably, the higher *T*_g_ of PS, P2VP, and PVN will influence the rigid P3HT interchain packing and crystallinity of the BCPs. In contrast, the low *T*_g_ and oil‐like nature of PBA will easily alter the packing pattern of the P3HT block and promote the mechanical properties of the derived BCP.^[^ ^28^ ^]^

Next, the optical characteristics of P3HT‐*b*‐PS, P3HT‐*b*‐P2VP, P3HT‐*b*‐PVN, P3HT‐*b*‐PBA, and the associated homopolymers were recorded by UV–vis absorption spectra (Figure 1c; Figure S4, Supporting Information). The optical bandgaps ($E_{g}^{\mathrm{opt}}$) of these BCPs extracted from the absorption onset of the UV–vis spectra are summarized in Table S1 (Supporting Information). All of the BCP films exhibited absorption bands across 337−702 nm with maximal absorbances at 525−559 nm and vibrational coupling peaks of 596−606 nm. These features were similar to that of P3HT homopolymer, indicating that solid‐state aggregation was well maintained even with the coexistence of insulating blocks. In addition, P3HT‐*b*‐P2VP and P3HT‐*b*‐PVN also possess UV absorption bands spanning the range of 232−277 nm, relating to the absorption of P2VP and PVN. As a result, these BCPs could possess panchromatic optical absorption covering the UV region to the red light region, and the resulting multiband photoresponse would be conducive to diverse applications of the phototransistor device. The steady‐state photoluminescence (PL) measurement was performed and is presented in Figure 1c. These BCPs showed an emission peak at 693 nm, which is the typical PL emission for P3HT with an excitation wavelength of 405 nm light. Photoinduced excitons have been known to play a nontrivial role in photonic transistor devices.^[^ ^29^, ^30^ ^]^ Next, the highest occupied molecular orbital (HOMO) levels for the P3HT‐based BCPs and insulating homopolymers were measured using cyclic voltammetry (CV), as shown in Figure S5 (Supporting Information), and the resultant HOMO and the lowest unoccupied molecular orbital (LUMO) levels are summarized in Figure S6 and Table S1 (Supporting Information). The energy levels (HOMO, LUMO) of the insulating homopolymers were (−1.82, −5.61), (−1.69, −6.15), (−1.66, −6.22), and (−1.73, −6.53) eV for PVN, P2VP, PS, and PBA, respectively. Favorable energy level adaptation has been known to be conducive to efficient charge transfer and promotes the performance of phototransistor devices.^[^ ^6^, ^31^ ^]^

To probe the microscopic influence of the different insulating blocks on their morphologies, the surface morphology of these BCPs was first investigated using AFM, and Figure S7a−d (Supporting Information) and Figure 1d–g present the surface AFM images for the as‐cast and thermally annealed thin films. A featureless morphology was observed from the as‐cast BCP films. After annealing the films, clear morphological transitions can be observed for P3HT‐*b*‐PS, P3HT‐*b*‐P2VP, and P3HT‐*b*‐PVN with the formation of nanoclusters, and for P3HT‐*b*‐PBA with the formation of nanofibrils. Notably, the nanofibrillar morphology of P3HT‐based BCPs is reported to ensure high carrier transport capability.^[^ ^32^ ^]^ The morphological transitions were related to the chain mobility of insulated polymers represented by their different *T*_g_ values.^[^ ^26^ ^]^ P3HT‐*b*‐PBA with a low *T*_g_ value below room temperature are highly mobile to form a confined microphase separation with P3HT wrapped inside the PBA matrix. However, P3HT‐*b*‐PS, P3HT‐*b*‐P2VP, and P3HT‐*b*‐PVN with high *T*_g_ values have difficulty in forming a nanofibrillar morphology, and therefore, remain a nanocluster morphology after thermal annealing.

The in‐depth molecular stacking and crystallographic properties of the BCP films were next investigated using GIWAXS analysis. Figure S7e−h (Supporting Information) and Figure 1h−k display the 2D GIWAXS patterns of the as‐cast and annealed BCP films, and Figure S8a,b (Supporting Information) presents the 1D GIWAXS profiles along the out‐of‐plane direction. All of these P3HT‐based BCPs present low‐*q* (*n*00) peaks in the out‐of‐plane direction, and the diffraction intensities of these BCP films are increased after thermal annealing. These packing patterns indicate that the BCP films possessed a typical edge‐on orientation. The lamellar spacing of these BCPs were derived by the following equation: *d* = 2*π*/*q*, where *q* is the position of out‐of‐plane (100) diffraction, and the corresponding *q* values are of 0.379, 0.387, 0.335, and 0.381 Å^−1^ for P3HT‐*b*‐PS, P3HT‐*b*‐P2VP, P3HT‐*b*‐PVN, and P3HT‐*b*‐PBA, respectively, which indicate the lamellar spacing of 1.66, 1.62, 1.88, and 1.65 nm for P3HT‐*b*‐PS, P3HT‐*b*‐P2VP, P3HT‐*b*‐PVN, and P3HT‐*b*‐PBA, respectively. In addition, the BCPs with stiffer insulating blocks, such as P3HT‐*b*‐PS, P3HT‐*b*‐P2VP, and P3HT‐*b*‐PVN, were observed to exhibit a higher crystallinity than that of P3HT‐*b*‐PBA. Accordingly, the relative degree of crystallinity of BCP films were calculated based on the out‐of‐plane diffraction according to a reported method with the baseline correction and intensity normalization.^[^ ^33^, ^34^ ^]^ To avoid the interference from the incident beam, the (200) diffraction is used to fairly compare their crystallinity, and the results are shown in Figure S8c,d (Supporting Information). These BCP films presented increased crystallinity from P3HT‐*b*‐PBA, P3HT‐*b*‐PS, P3HT‐*b*‐PVN to P3HT‐*b*‐P2VP, and this trend is relating to the phase transition points of the insulating blocks. As mentioned earlier, the lower *T*_g_ and oil‐like nature of PBA among insulating blocks in all the BCP samples will easily alter the packing pattern of the P3HT block, leading to the lowest crystallinity of P3HT‐*b*‐PBA. In contrast, PS, PVN, and P2VP, owing to their high *T*_g_s at room temperature, ensure their integrity in solid‐state stacking, resulting in much higher crystallinity of the resultant BCPs than P3HT‐*b*‐PBA. In particular, P3HT‐*b*‐PS with a lower *T*_g_ than P3HT‐*b*‐PVN and P3HT‐*b*‐P2VP presented greater mobility of the molecular chains and thereby possessed somewhat lower crystallinity.^[^ ^35^ ^]^ It is understood that P3HT with an ordered structure is conducive to carrier transport, and the structure−performance relationship of these BCPs as active channels in phototransistor devices was subsequently investigated.

### 2.2 Charge Dissociation Dynamics and Transient Photocurrent Characteristics

**Figure 2:**
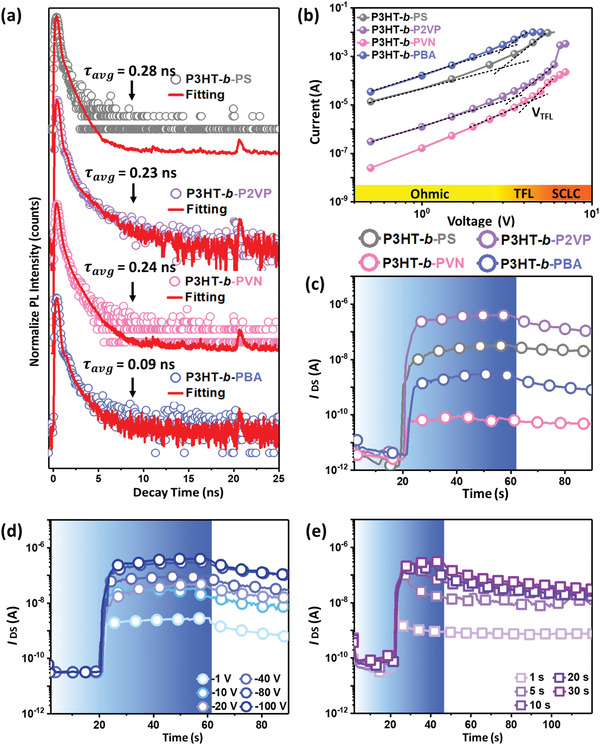
a) TR‐PL profiles and the fitting curves of the P3HT‐based BCP films. b) The quantization of trap density and mobility of P3HT‐based BCPs by SCLC measurement. c) Temporal *I*_DS_ curves of the phototransistors comprising the thermally annealed BCP films with photowriting of 405 nm, 10 mW cm^−2^ for 40 s at *V*_DS_ of −100 V. Temporal *I*_DS_ curves of the phototransistor device comprising the thermally annealed P3HT‐*b*‐P2VP film at d) varied *V*_DS_ and illumination time of 40 s and e) varied illumination time and *V*_DS_ of −100 V. The color regions in the temporal *I*_DS_ curves represent the light illumination.

To gain insight in the charge dissociation dynamics of the P3HT‐based BCPs, time‐resolved photoluminescence (TR‐PL) analysis, as a persuasive method to describe the dynamics of nonradiative and radiative decay pathways of the photoexcited carriers, was applied to these BCP films. The fluorescence decay curves are displayed in **Figure** **2**a, and the carrier lifetime (*τ*) is fitted using a reconvolution biexponential function, as expressed in Equation (1)

(1) $$I\left(t\right)=\int_{-\infty }^{t}\mathrm{IRF}\;\left(t^{\prime }\right)\sum_{i\;=\;1}^{n}A_{i}e^{\frac{-\left(t-t^{\prime }\right)}{\tau_{i}}}dt^{\prime }$$

where *A_i_* is the corresponding weight factor, *τ*_1_ is the fast decay component that is attributed to the nonradiative recombination in the P3HT domains, and *τ*_2_ is the slow decay component that is related to the radiative recombination of free carriers.^[^ ^36^ ^]^ The relevant fitting parameters and the intensity weighted average carrier lifetime (*τ*_avg_) are summarized in Table S2 (Supporting Information). The *τ*_avg_ values of P3HT‐*b*‐PS, P3HT‐*b*‐P2VP, P3HT‐*b*‐PVN, and P3HT‐*b*‐PBA were 0.28, 0.23, 0.24, and 0.09 ns, respectively, affirming the photogenerated excitons in the nanoclusters of P3HT‐*b*‐PS, P3HT‐*b*‐P2VP, and P3HT‐*b*‐PVN are easier transferred and less recombined than those in the nanofibrils of P3HT‐*b*‐PBA. To further understand the stability of the photogenerated charge on different insulating blocks, we quantize the trap density (*N*_trap_) and carrier mobility (*μ*_SCLC_) of P3HT‐based BCP films. Figure 2b depicts the space‐charge‐limited‐current (SCLC) curves, where three distinct regions can be defined. The first region is the linear region of current and voltage that obeys Ohm's law. The second region is the nonlinear trap‐filled limit (TFL) region, for which the trap sites are filled by the injected charges. The trap‐filled limit voltage (*V*_TFL_) is located between the ohmic and trap‐filled regimes, and the *N*_trap_ can be calculated using Equation (2)^[^ ^37^ ^]^

(2) $$N_{\mathrm{trap}}=\frac{2\varepsilon_{0}\varepsilon_{r}}{e}\frac{V_{\mathrm{TFL}}}{L^{2}}$$

where *ε*_0_ is the vacuum permittivity, *ε*_r_ is the dielectric constant, *L* is the thickness of a BCP film, and *e* is the elementary charge. The third region is Child's regime, in which the current increases quadratically as the voltage increases. Assuming all the traps are filled by carriers, the *μ*_SCLC_ can be estimated by the Mott–Gurney Equation (3)^[^ ^38^ ^]^

(3) $$\mu_{\mathrm{SCLC}}=\frac{8}{9}\;\frac{J_{D}}{\varepsilon_{0}\varepsilon_{r}}\frac{L^{3}}{V^{2}}$$

where the *J*_D_ is the current density in the dark. The dielectric constant, trap‐filled limit voltage, trap density, and mobility of P3HT‐based BCPs are summarized in Table S3 (Supporting Information). Accordingly, P3HT‐*b*‐P2VP possesses the lowest trap density of 4.9 × 10^24^ m^−3^ and the highest mobility of 1.3 × 10^−8^ cm^2^ V^−1^ s^−1^. It is understood that a lower trap density and higher mobility is favorable for preventing recombination of the photogenerated electrons/holes^[^ ^39^ ^]^, and this result is in a good relevance to the findings in the TR‐PL analysis. Combining the results from TR‐PL and SCLC analyses, P3HT‐*b*‐P2VP is regarded as a high‐performance channel material for phototransistor application.

### 2.3 Memory Behavior Characteristics of the Electret‐Free Phototransistors

Phototransistor memory with a typical bottom‐gate/top‐contact architecture and using these P3HT‐based BCPs as the channel materials were fabricated. The transfer curves of the phototransistors were recorded by sweeping the gate voltage (*V*_GS_) between 20 and −100 V under a fixed drain voltage (*V*_DS_) of −100 V, are displayed in Figure S9a–d (Supporting Information). Device parameters, including the field‐effect mobility (*μ*_h_), ON/OFF current ratio (*I*_ON_/*I*_OFF_), and memory windows (Δ*V*_th_), are listed in Table S4 (Supporting Information). *μ*_h_ was estimated from the slope of the square root of the drain current (*I*_DS_), the *I*_ON_/*I*_OFF_ was derived from the variations of *I*_DS_ at *V*_GS_ = 0 V after photowriting and electrical erasing processes, and Δ*V*_th_ was defined as the difference between the threshold voltage (*V*_th_) in the photowriting and electric erasing states. The memory performance was affected by the operating voltage of *V*_GS_ and *V*_DS_, light wavelength and intensity, and the illumination time. For a fair comparison, the transfer characteristics were conducted at a fixed *V*_DS_ = −100 V with same light sources and illumination time of 40 s. As seen in Figure S9 (Supporting Information), the transfer curves distinctly shifted to the positive region after photowriting with 254, 405, 530, and 650 nm light. The *V*_th_ shifts is related to the formation of a built‐in electric field opposite to the *V*_th_ shift, which indicates the electron trapping propensity in the BCP channel. After applying *V*_GS_ of −100 V for 1 s, the transfer curves returned to their initial positions, and a clear *I*_ON_/*I*_OFF_ value could be defined from the photowriting and electric erasing states. For example, P3HT‐*b*‐P2VP shows Δ*V*_th_s of 38, 31, 29, and 27 V after photowriting with 254, 405, 530, and 650 nm light, and this disparity is related to three aspects: i) phonon energy of the incident light, ii) light intensity, and iii) optical absorbance of the polymer at the corresponding wavelength. The low‐intensity 254 nm light has the highest phonon energy in the applied light, and therefore, produces a pronounced modulation in the device photocurrent. In contrast, P3HT‐*b*‐P2VP shows a relatively low photoresponse to 650 nm light owing to the weak absorbance and low phonon energy of red light.

To gain insight into the transient characteristics of the phototransistors, photowriting operation with varied light wavelengths for 40 s and the subsequent relaxation of *I*_DS_ was tracked at *V*_DS_ of −100 V and *V*_GS_ of 0 V. The temporal *I*_DS_ curves of the devices are displayed in Figure 2c, and Figure S10 (Supporting Information) for P3HT‐*b*‐P2VP and other BCPs‐based devices. As can be seen, the devices present clear transitions from the low conductance states (<10^−11^ A) to the high conductance (P3HT‐*b*‐PS: 3.1 × 10^−8^ A, P3HT‐*b*‐P2VP: 3.9 × 10^−7^ A, P3HT‐*b*‐PVN: 6.9 × 10^−11^ A, and P3HT‐*b*‐PBA: 3.1 × 10^−9^ A) after photowriting with 405‐nm light for 40 s. After removing the light, the photocurrents are highly remained, which indicates their nonvolatile memory property. The thermally annealed BCP films exhibit decent *I*_ON_/*I*_OFF_ values, possibly due to the nanocluster morphology and the promoted crystallinity, which are conducive to provide more efficient carrier transport without interfering with the charge storage inside the insulating block domains. In addition, the photodynamics of BCPs and the energy level alignment between the constituent polymers also play an important role in the device performance. Notably, P3HT‐*b*‐P2VP exhibited a much higher *I*_ON_/*I*_OFF_ of 10^5^, outperforming other BCP analogs. This trend was also clearly observed with varied applied light, including UV (254 nm), green (530 nm), and red (650 nm) light. In particular, P3HT‐*b*‐P2VP presented the utmost improvement in the photoresponse to UV light, showing a high *I*_ON_/*I*_OFF_ of 10^5^. The high performance of P3HT‐*b*‐P2VP is strongly relating to the favorable morphology and energy level alignment associated with its long exciton lifetime.

To evaluate the efficacy of BCP design in the device performance, the reference device with P3HT homopolymer as a channel was fabricated and characterized. Figure S11a (Supporting Information) depicts the temporal *I*_DS_ curves of the reference device with 405 nm light illumination. As can be seen, P3HT presents a fast photoresponse and a poor retention on the photocurrent, which shows a low *I*_ON_/*I*_OFF_ of 10^1^ after removing the light. This result clearly indicates the BCP design on P3HT is conducive to improve the charge stability in a memory device without an electret.

Given the high performance of P3HT‐*b*‐P2VP, its voltage‐dependent and illumination time‐dependent transient characteristics were subsequently studied. Figure 2d illustrates the temporal *I*_DS_ curves of the phototransistor device with varied *V*_DS_ levels from −1 to −100 V under illumination of blue light for 40 s. It is worth noting that a low *V*_DS_ of −1 V was sufficient to achieve an *I*_ON_/*I*_OFF_ of 10^2^–10^3^. In addition, the P3HT‐*b*‐P2VP‐based device was tested with different illumination times (1−30 s) with blue light to explore the efficacy of the fast photoresponse. As seen in Figure 2e, P3HT‐*b*‐P2VP could achieve a high *I*_ON_/*I*_OFF_ of 10^3^–10^4^ and 10^2^ after blue light illumination for 10 and 1 s, respectively. By manipulating these parameters, well‐defined memory states were demonstrated with the P3HT‐*b*‐P2VP‐based device, and this property is conducive to application in shrinking image capturing, recording, and logic data processing.

With regard to the device stability, their long‐term stability and ambient operation were tested. As can be seen in Figures S12 (Supporting Information), the phototransistor memory comprising the P3HT‐based BCPs produces similar photoresponses after storing in nitrogen atmosphere for 8 months. In addition, these devices provide similar performances in nitrogen atmosphere and ambient condition (Figure S13, Supporting Information). It is understood that BCP design on P3HT with an insulating coil could be conducive with the environmental stability.^[^ ^40^ ^]^

The long‐term retention capability of the memory behavior with P3HT‐*b*‐P2VP film was tested by monitoring the *I*_DS_ at a fixed *V*_DS_ = −100 V and *V*_GS_ = 0 V in the dark condition for the OFF‐state current, and the device was photowritten by illumination of UV, blue, green, and red light for 40 s for the ON‐state current. Figure S14 (Supporting Information) represents the long‐term stability of P3HT‐*b*‐P2VP, showing high *I*_ON_/*I*_OFF_ of 10^5^, 10^5^, 10^3^, and 10^2^ to UV, blue, green, and red light over 10^4^ s, affirming its outstanding retention capability and multistate memory applications. The memory endurances of these BCP films were next examined. Figure S15 (Supporting Information) displays the endurance measurements of these devices operated at a fixed *V*_DS_ of −100 V while the photowriting (405 nm light) and reading procedures were conducted at *V*_GS_ = 0 V, and the electrical erasing procedure was applied by using *V*_GS_ of −100 V for 1 s. The P3HT‐*b*‐P2VP based device achieved a high *I*_ON_/*I*_OFF_ of 10^5^ during the WRER operation, outperforming P3HT‐*b*‐PS, P3HT‐*b*‐PVN, and P3HT‐*b*‐PBA, which showed *I*_ON_/*I*_OFF_ values of < 10^3^.

**Figure 3:**
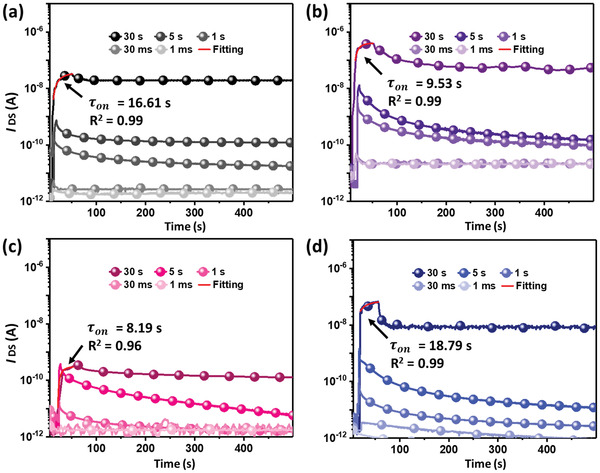
Transient photocurrent characteristics of the phototransistors comprising the thermally annealed a) P3HT‐*b*‐PS, b) P3HT‐*b*‐P2VP, c) P3HT‐*b*‐PVN, and d) P3HT‐*b*‐PBA films photowritten with different illumination time (450 nm light; 22.0 mW cm^−2^) from 1 ms to 30 s at *V*_DS_ of −100 V.

Next, the transient photocurrent characteristics spanning the range of 1−30 ms were conducted to evaluate their photoresponse and STM capability after an ultrashort light illumination. **Figure** **3**a–d displays the characteristics for P3HT‐*b*‐PS, P3HT‐*b*‐P2VP, P3HT‐*b*‐PVN, and P3HT‐*b*‐PBA. Notably, P3HT‐*b*‐PS and P3HT‐*b*‐P2VP show good *I*_ON_/*I*_OFF_ of 2.6 and 7.7 after ultrashort light illumination for 1 ms. To quantify the transient dynamics of photogenerated carriers, their photocurrent curves after light illumination for 30 s are fitting by Equation (4)

(4) $$I=A\left[1-\exp\left[\frac{-t}{\tau_{\mathrm{on}}}\right]\right]+I_{\mathrm{dark}}$$

where *t*, *τ*_on_, *A*, and *I*_dark_ is the time, characteristic‐ON time, scaling factor and dark current. The *τ*_on_ of P3HT‐*b*‐PS, P3HT‐*b*‐P2VP, P3HT‐*b*‐PVN, and P3HT‐*b*‐PBA are 16.6, 9.5, 8.2, and 18.8 s, respectively. A low *τ*_on_ indicates the photogenerated excitons are easier transferred and less recombined. As can be seen, photogenerated excitons in the nanoclusters of P3HT‐*b*‐P2VP, and P3HT‐*b*‐PVN are easier transferred and less recombined than those in the nanofibrils of P3HT‐*b*‐PBA, and this result is in good relevance to the characteristic carrier lifetime in TR‐PL profiles.

### 2.4 Synaptic Behavior Characteristics of the Electret‐Free Phototransistors

**Figure 4:**
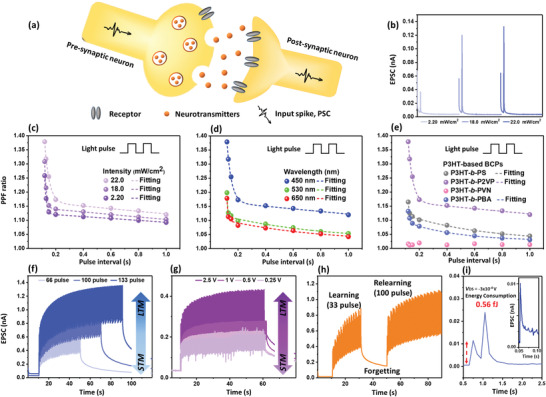
a) Schematic illustration of the artificial synapse. b) EPSC of the photosynaptic transistor device comprising P3HT‐*b*‐P2VP giving different intensity of 450 nm light pulses with a pulse width of 0.2 s. PPF ratios of the photosynaptic transistor device comprising P3HT‐*b*‐P2VP with c) different intensity of 450 nm light pulses or d) different wavelength of light with an intensity of 22.0 mW cm^−2^. Note that the photosynaptic transistors were operated at *V*_DS_ = −5 V. e) Performance comparison of the device comprising different BCPs. f) Pulse number dependent and g) operating voltage dependent EPSC curves with 84 light pulses of the photosynaptic transistor device comprising P3HT‐*b*‐P2VP. h) Learning/forgetting process of P3HT‐*b*‐P2VP‐based photosynaptic transistor operated at *V*_DS_ = −5 V. i) Energy consumption of the photosynaptic transistor operated at *V*_DS_ = −0.0003 V. Note that the EPSC curves in (f)−(i) were tracked by giving the presynaptic light pulses with a width of 0.2 s, light intensity of 2.2 mW cm^−2^, and light wavelength of 450 nm.

We further demonstrate the efficacy of these phototransistor devices in the appreciation of artificial synapses by using fast light stimuli. P3HT‐based BCPs were capable of simulating communication transmission in neuromorphic architectures. Synapses comprise presynaptic and postsynaptic sites and the synaptic gap between them.^[^ ^41^ ^]^ The fundamental structure of photonic synapses is similar to the biological synapses, the source/drain electrodes correspond to presynaptic and postsynaptic sties, light pulses are employed to stimulate the potentials, and the channels between the two electrodes are equivalent to neurotransmitters, as depicted in **Figure** **4**a. When these neurotransmitters diffuse from presynaptic to postsynaptic sites, they generate the excitatory postsynaptic current (EPSC) or the inhibitory postsynaptic current (IPSC), which is relating to the synapse strength.

In view of the photosynaptic transistor comprising the P3HT‐based BCPs, the photogenerated carriers in the P3HT photogates subsequently recombine with the stored charges, and this process is similar to the behavior of a biological synapse during light illumination. The learning and forgetting processes in the human brain are mimicked by the accumulation and loss of EPSC levels triggered by light pulses. Figure 4b displays the EPSC variation of the photosynaptic transistor comprising P3HT‐*b*‐P2VP stimulated by controlling the intensity of blue light at a wavelength of 450 nm. Increases in the EPSC of 0.038, 0.121, and 0.132 nA were achieved by applying one light pulse with intensities of 2.20, 18.0, and 22.0 mW cm^−2^, respectively. A higher change in EPSCs indicates stronger synaptic plasticity to emulate recognition, learning, and memory in the human brain. To quantify the enhancement in EPSCs, paired‐pulse facilitation (PPF) was introduced as an important factor of short‐term plasticity with two consecutive presynaptic pulses. PPF can be described as follows^[^ ^42^ ^]^

(5) $$\mathrm{PPF}=\frac{A_{2}}{A_{1}}\times 100\%$$

where *A*_1_ and *A*_2_ are the first and second EPSCs. The PPF fitting results are displayed in Table S5 (Supporting Information), and a longer characteristic time indicates better synaptic behavior. As the pulse interval increases, the PPF index gradually decreases. Note that the electret‐free photosynaptic transistor was operated without a gate bias, that is, in a photoconductive stage, which promoted the device photocurrent and responsivity than that of a photovoltaic stage.^[^ ^43^, ^44^ ^]^

Next, the photosynaptic transistor was tested with different intensities of 450 nm light (Figure 4c) or different light wavelengths with an intensity of 22.0 mW cm^−2^ (Figure 4d). The PPF index can be fitted by two exponential decay curves as follows^[^ ^45^ ^]^

(6) $$\mathrm{PPF}=c_{1}\;\exp\left(-\frac{\Delta t}{\tau_{1}}\right)+c_{2}\exp\left(-\frac{\Delta t}{\tau_{2}}\right)$$

where *τ*_1_ and *τ*_2_ are the characteristic relaxation of the slow and rapid phases. The PPF index increased from 1.26, 1.31, to 1.38 as the light intensities were increased from 2.20, 18.0, to 22.0 mW cm^−2^, respectively. P3HT‐*b*‐P2VP delivers the highest PPF index to blue light compared to green (1.20) and red light (1.18). In addition, other BCPs, including P3HT‐*b*‐PS, P3HT‐*b*‐PVN, and P3HT‐*b*‐PBA, were tested similarly with a 450 nm light pulse (22.0 mW cm^−2^), and their relationship between the PPF index and pulse intervals is summarized in Figure 4e. The maximum PPF indices of P3HT‐*b*‐PS, P3HT‐*b*‐P2VP, and P3HT‐*b*‐PBA were 1.16, 1.38, and 1.13, respectively, and P3HT‐*b*‐PVN did not possess synaptic behavior. As mentioned before, P3HT‐*b*‐P2VP with a high crystallinity and a favorable energy level adaptation with P3HT are conducive to forming a high‐performance photosynaptic transistor.

Next, to evaluate the effect of BCP design on the device performance, the reference synaptic device with P3HT homopolymer as a channel was tested similarly with a 450 nm light pulse (22.0 mW cm^−2^). Figure S11b (Supporting Information) depicts the relationship between the PPF index and pulse intervals of the P3HT‐based device. As can be seen, P3HT presents a lower PPF index of 1.05 than that of 1.37 from P3HT‐*b*‐P2VP. This result clarifies the importance of BCP design on the performance of a synaptic transistor without an electret. Compared to the photosynaptic transistor with P3HT as a channel reported by Li et al., the device presented a high PPF index of 1.4, and they attributed the synaptic behavior of the device to interfacial traps between the P3HT channel and SiO_2_ dielectric. When a (3‐aminopropyl) trimethoxylsilane self‐assembled monolayer (SAM) was introduced between the channel and dielectric layers, the EPSC of the device enhanced and decayed faster than that without the hydrophilic SAM.^[^ ^46^ ^]^ In this study, the contribution from interfacial traps is precluded by an octadecyltrimethoxysilane (ODTS) SAM between the channel and dielectric layers. Therefore, the P3HT‐based device presented a low photoresponse, and the synaptic performance of BCP‐based devices was presumably occurred in the channel.

Generally, LTM in the human brain can accumulate from STM through repeated rehearsal events. Figure 4f depicts the synaptic transition process from STM to LTM of the photosynaptic transistor comprising P3HT‐*b*‐P2VP by controlling the number of light pulses. The PSC gradually increases with an increased pulse number from 66 to 133. As the light stimulus is stopped, the PSC gradually decreases with varied plasticity. This phenomenon indicates the transitions from STM to LTM similar to the human brain. In addition, the STM to LTM transitions could be emulated by varying the operating *V*_DS_ from −2.5 to −0.25 V (Figure 4g).

When human beings receive new information, a series of learning−forgetting−relearning processes is conducted to perpetuate the knowledge. With the plasticity endowed by pulse number‐ and voltage‐dependent operations, artificial synapses can perform similarly to learning−forgetting−relearning processes. For example, the device was imposed with 33 consecutive light pulses at *V*_DS_ = −5 V and showed an increased EPSC. Next, the light stimulus was removed, and the device presented a decreased EPSC. Finally, the EPSC level rapidly reached the previous EPSC maximal level with 10 light pulses and continuously increased with the following 100 light pulses. The respective variation of the EPSC levels in the learning−forgetting−relearning processes is displayed in Figure 4h. After demonstrating the emulation of the learning process, the energy consumption is next evaluated owing to its great importance to fulfill a low‐power neuromorphic computation system. The energy consumption (*E*) of a synaptic transistor can be calculated by using the following equation

(7) $$E=I_{\mathrm{peak}}\times t\times V$$

where *I*_peak_, *t*, and *V* are the peak value of EPSC, light pulse width, and operating voltage, respectively.^[^ ^47^ ^]^ As seen in Figure 4i, taking the EPSC variations at *V*_DS_ = −0.0003 V, the photosynaptic transistor comprising P3HT‐*b*‐P2VP presented an ultralow *E* of 0.56 fJ. This achievement is comparable to the reported electret‐free photosynaptic transistors spanning the range of 0.03−17.5 fJ as shown in **Table** **1**.^[^ ^25^, ^48^, ^49^, ^50^, ^51^, ^52^, ^53^, ^54^ ^]^ It is understood that an electret‐free phototransistor is capable of forming highly sensitive photodetectors and low‐energy‐consumption artificial synapses, having high volatilities and low hysteresis. In contrast, a floating gate phototransistor produces higher stability in the trapped charges than that of an electret‐free device, and therefore, a floating gate phototransistor is favorable for nonvolatile memory application.^[^ ^55^, ^56^ ^]^ With the dual functions of nonvolatile memory and low‐energy‐consumption artificial synapses, the phototransistor device comprising P3HT‐based BCPs was believed to show great impact on the photocommunication community.

**Table 1:** Device parameters of the electret‐free photosynaptic transistors in the literatures

Device	Wavelength [nm]	Light intensity [mW cm^−2^]	Operating voltage [V]	Energy consumption	Ref.
C8‐BTBT^a)^	360	0.9	−1	420 pJ	^[^ ^22^ ^]^
Si NCs^b)^	532	1.3	5 × 10^−2^	140 pJ	^[^ ^48^ ^]^
PQT‐12/CsPbBr_3_ QD^c)^	500	100	−1	650 pJ	^[^ ^49^ ^]^
SWCNT/chlorophyll^d)^	665	0.5	−10^−4^	17.5 fJ	^[^ ^50^ ^]^
PDPP4T/chlorophyll^e)^	430	N/A	−10^−5^	0.25 fJ	^[^ ^51^ ^]^
PDPPTT/CsPbBr_3_ QD^f)^	450	0.05	−5 × 10^−5^	0.5 fJ	^[^ ^52^ ^]^
P3HT/CsPbBr_3_ QD CNFs^g)^	450	10	−10^−3^	0.18 fJ	^[^ ^53^ ^]^
P3HT/FAPbBr_3_ QD^h)^	450	6.1	−5 × 10^−4^	0.03 fJ	^[^ ^54^ ^]^
P3HT‐*b*‐P2VP	450	2.2	−3 × 10^−4^	0.56 fJ	This work

^a)^ C8‐BTBT: 2,7‐dioctyl[1]benzothieno[32‐b][1]benzothiophene.

^b)^ Si NCs: silicon nanocrystals.

^c)^ PQT‐12: poly(3,3‴‐didodecyl[2,2′:5′,2″:5″,2‴‐quaterthiophene]‐5,5‴‐diyl).

^d)^ SWCNT: single‐wall carbon nanotube.

^e)^ PDPP4T: poly[2,5‐bis(2‐octyldodecyl)pyrrolo[3,4‐c]pyrrole‐1,4(2H,5H)‐dione‐3,6‐diyl)‐*alt*‐(2,2′;5′,2″;5″,2‴‐quaterthiophen‐5,5‴‐diyl)].

^f)^ PDPPTT: poly[2,5‐(2‐octyldodecyl)‐3,6‐diketopyrrolopyrrole‐*alt*‐5,5‐(2,5‐di(thien‐2‐yl)thieno[3,2‐b] thiophene)].

^g)^ CNFs: composite nanofibrils of P3HT and CsPbBr_3_ QD.

^h)^ FAPbBr_3_: formamidinium lead bromide.

### 2.5 Structure−Performance Relationship and Proposed Mechanism for the Electret‐Free Phototransistor with P3HT‐Based BCPs

The structure−performance relationship of these P3HT‐based BCPs relies on three aspects: i) rigidity of the insulating blocks, ii) energy levels between the insulating blocks and P3HT, and iii) electrophilicity of the functional groups on the insulating blocks. In view of the rigidity, as mentioned before, P3HT‐*b*‐PBA with a low *T*_g_ easily formed confined nanostructures, and P3HT was wrapped inside the PBA matrix. However, the varying degrees of *T*_g_ of P3HT‐BCPs can influence the morphology (nanoclusters and nanofibrillar), crystallinity, and charge trapping ability. Consequently, P3HT‐*b*‐PVN and P3HT‐*b*‐P2VP could render an ordered domain of P3HT for high carrier transporting capability, and the nanocluster morphology engendered efficient exciton separation and fast charge trapping.

**Figure 5:**
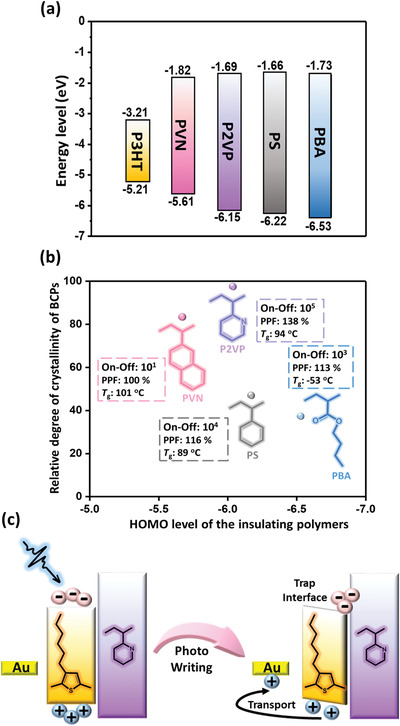
a) Energy level diagram of the constituent homopolymers. b) Structure−performance relationship of the low‐energy‐consumption and electret‐free photosynaptic transistor with P3HT‐based BCPs as channels. c) Operating mechanism of the electret‐free phototransistor device.

In view of the energy level alignment between P3HT and the insulating homopolymers (**Figure** **5**a), P3HT‐*b*‐PVN and P3HT‐*b*‐P2VP possessed HOMO levels closer to P3HT than P3HT‐*b*‐PS and P3HT‐*b*‐PBA, and this disparity is relating to the conjugation of the functional group on the insulating blocks. The photoinduced exciton will accumulate at the P3HT block during light illumination, and the photogenerated electrons are transferred to the P3HT/insulating segments interfaces, and holes are prone to transfer through the P3HT segment only if their HOMO levels are close enough. With a favorable energy level alignment, the photoinduced excitons are enabled to smoothly transduce into electron/hole pairs with prolonged exciton lifetime in the presence of PVN, P2VP, and PS, and holes are capable of transferring to the source/drain electrodes while electrons are trapped inside the channel. In contrast, the deeper‐lying HOMO level and short exciton lifetime of P3HT‐*b*‐PBA are not favorable for effective charge transfer. However, the very close HOMO levels between P3HT and PVN will eventually induce hole back‐trapping and charge recombination, giving rise to the low mobility and weak photoresponse of P3HT‐*b*‐PVN.

In view of the electrophilicity of the functional groups on the insulating blocks, pyridine, as a *π*‐electron‐deficient moiety, similar to quinoxaline and thiadiazole with an electron‐withdrawing imine (—C═N—) structure, is conducive to electron trapping.^[^ ^57^ ^]^ In contrast, styrene and butyl acrylate are not *π*‐electron‐deficient moieties for effective electron trapping.^[^ ^58^ ^]^ Naphthalene with a higher conjugation is capable of trapping both electrons and holes and is prone to induce charge recombination.^[^ ^59^ ^]^ Finally, the structure−performance relationship of these P3HT‐based BCPs is summarized in Figure 5b. Insulating polymer with a deeper‐lying HOMO level compared to P3HT, and a suitable *T*_g_ of the insulating segment is conducive to achieving high‐performance BCP in an electret‐free photonic transistor device. Finally, we hypothesize a plausible mechanism of the device comprising P3HT‐*b*‐P2VP, as illustrated in Figure 5c. During light stimulation, the accumulated excitons transfer from the P3HT block to the interfaces of the P2VP block, smoothly transducing into electron and hole pairs in the presence of P2VP. Then, holes are enabled to transfer to the channel electrodes, and electrons are trapped inside the channel. This result is relating to the increased photocurrent, and the homogeneity of BCP is attributed to the ultrafast photoresponse of the phototransistor. Based on the above discussions, P3HT‐*b*‐P2VP is therefore regarded as a sought‐after combination to produce the best synaptic performance among the BCPs studied.

## 3 Conclusion

In conclusion, a series of P3HT‐based BCPs were applied in electret‐free phototransistors to evaluate their STM/LTM capability and synaptic behaviors. We found that the structure–performance relationship of these P3HT‐based BCPs highly relies on three aspects: i) rigidity of the insulating blocks, ii) energy levels between the insulating blocks and P3HT, and iii) electrophilicity of the functional groups on the insulating blocks. P2VP with a deeper‐lying HOMO level than P3HT and a suitable *T*_g_ is conducive to forming a high‐performance electret‐free phototransistor showing long‐term memory and synaptic behaviors. Accordingly, P3HT‐*b*‐P2VP‐based photosynaptic transistor with a sought‐after BCP combination demonstrated LTM behavior with current contrast up to 10^5^, STM behavior with high PPF ratio of 1.38, and an ultralow energy consumption of 0.56 fJ at an operating voltage of −0.0003 V. Our findings in this study provide a new perspective on material designs for electret‐free phototransistor device.

## 4 Experimental Section

### Materials

Poly(3‐hexylthiophene)‐*block*‐poly(styrene) (P3HT‐*b*‐PS, *M*_n_ (P3HT) = 10 000, *M*_n_ (PS) = 13 500, *Ð*_M_ = 4.5) was purchased from Polymer Source, Inc. (Quebec, Canada). Poly(3‐hexylthiophene)‐*block*‐poly(butyl acrylate) (P3HT‐*b*‐PBA, *M*_n_ (P3HT) = 6000, *M*_n_ (PBA) = 6000, *Ð*_M_ = 1.15) was synthesized according to our previous work through copper‐catalyzed azide–alkyne cycloadditions with alkyne‐functionalized P3HT and azide‐functionalized PBA.^[^ ^27^ ^]^ Syntheses of poly(3‐hexylthiophene)‐*block*‐poly(2‐vinylpyridine) (P3HT‐*b*‐P2VP, *M*_n_ (P3HT) = 8000, *M*_n_ (P2VP) = 7100, *Ð*_M_ = 1.14) and poly(3‐hexylthiophene)‐*block*‐poly(2‐vinylnaphthalene) (P3HT‐*b*‐PVN, *M*_n_ (P3HT) = 5500, *M*_n_ (PVN) = 5200, *Ð*_M_ = 1.15) were detailed in the Supporting Information. The ^1^H‐NMR spectra in CDCl_3_ or C_2_D_2_Cl_4_ and SEC profiles in THF of P3HT‐*b*‐P2VP and P3HT‐*b*‐PVN were presented in Figures S1 and S2 (Supporting Information).

### Fabrication and Characterization of the Phototransistor

The phototransistor was fabricated based on a typical bottom‐gate/top‐contact architecture of Si/SiO_2_/BCP/Au configuration. A 300 nm thick SiO_2_ layer (capacitance per unit area = 10 nF cm^−2^) as a gate dielectric was thermally grown onto the highly n‐type doped Si(100) substrates with an ODTS SAM modification on the SiO_2_ layer. The BCP solution in chloroform (3 mg mL^−1^) was heated at 60 °C for 6 h, filtered through a PTFE syringe filter with a pore size of 0.22 µm, and spin coated onto the ODTS‐modified SiO_2_/Si substrate at a spin rate of 2000 rpm for 60 s. The thickness of the BCP films were approximately 20−22 nm (P3HT‐*b*‐PS: 21.1 nm, P3HT‐*b*‐P2VP: 21.9 nm, P3HT‐*b*‐PVN: 21.7 nm, and P3HT‐*b*‐PBA: 20.6 nm) as shown in the surface profiles (Figure S16, Supporting Information). After that, the BCP film was thermally annealed at 150 °C under nitrogen atmosphere for 1 h. Finally, a 70 nm thick Au layer was thermally evaporated through a regular shadow mask with channel length (*L*) and width (*W*) of 25 and 1500 µm to pattern the top‐contact source and drain electrodes.

The memory characterization of phototransistors was characterized by using a Keithley 4200‐SCS semiconductor parameter analyzer (Tektronix) in a nitrogen‐filled glovebox at room temperature. The photowriting process was conducted with UV light (254 nm, 0.916 mW cm^−2^); blue light (405 nm, 10 mW cm^−2^); green light (530, 10 mW cm^−2^); red light (650 nm, 8 mW cm^−2^) for 40 s and electrical erasing with *V*_GS_ of −100 V for 1 s. The transfer curves and transient characteristics were collected at *V*_DS_ of −100 V. The photosynaptic transistors were characterized by using a Keithley 2634B in a nitrogen‐filled glovebox at room temperature with a monochromatic photonic source (Sadhu Design Co.) of 450 nm with tunable light intensity of 0.5−22 mW cm^−2^ and light interval of 0.12−1 s, which was calibrated by a laser power meter (Thorlabs PM 100D). All the measurements were conducted in darkness to prevent additional environmental light sources.

## Conflict of Interest

The authors declare no conflict of interest.

## Supporting information

Supporting Information

## Data Availability

The data that support the findings of this study are available from the corresponding author upon reasonable request.

## References

[1] C. Liu , X. Yan , X. Song , S. Ding , D. W. Zhang , P. Zhou , Nat. Nanotechnol. 2018, 13, 404. 29632398 10.1038/s41565-018-0102-6

[2] Z. Lv , M. Chen , F. Qian , V. A. L. Roy , W. Ye , D. She , Y. Wang , Z. X. Xu , Y. Zhou , S. T. Han , Adv. Funct. Mater. 2019, 29, 1902374.

[3] P. A. Merolla , J. V. Arthur , R. Alvarez‐Icaza , A. S. Cassidy , J. Sawada , F. Akopyan , B. L. Jackson , N. Imam , C. Guo , Y. Nakamura , B. Brezzo , I. Vo , S. K. Esser , R. Appuswamy , B. Taba , A. Amir , M. D. Flickner , W. P. Risk , R. Manohar , D. S. Modha , Science 2014, 345, 668. 25104385 10.1126/science.1254642

[4] M. M. Waldrop , Nat. News 2016, 530, 144. 10.1038/530144a 26863965

[5] W. C. Yang , Y. C. Chiang , J. Y. Lam , T. H. Chuang , E. Ercan , C. C. Chueh , W. C. Chen , Adv. Electron. Mater. 2020, 6, 2000458.

[6] W. C. Yang , Y. C. Lin , M. Y. Liao , L. C. Hsu , J. Y. Lam , T. H. Chuang , G. S. Li , Y. F. Yang , C. C. Chueh , W. C. Chen , ACS Appl. Mater. Interfaces 2021, 13, 20417. 33886254 10.1021/acsami.1c03402

[7] C. C. Shih , Y. C. Chiang , H. C. Hsieh , Y. C. Lin , W. C. Chen , ACS Appl. Mater. Interfaces 2019, 11, 42429. 31625392 10.1021/acsami.9b14628

[8] S. Yang , Y. Zhang , T. Wang , W. Sun , Z. Tong , ACS Appl. Mater. Interfaces 2020, 12, 46701. 32960035 10.1021/acsami.0c13531

[9] E. Ercan , J. Y. Chen , C. C. Shih , C. C. Chueh , W. C. Chen , Nanoscale 2018, 10, 18869. 30277243 10.1039/c8nr06396f

[10] M. Y. Liao , Y. C. Chiang , C. H. Chen , W. C. Chen , C. C. Chueh , ACS Appl. Mater. Interfaces 2020, 12, 36398. 32700518 10.1021/acsami.0c10587

[11] Q. Li , T. Li , Y. Zhang , Y. Yu , Z. Chen , L. Jin , Y. Li , Y. Yang , H. Zhao , J. Li , J. Yao , Org. Electron. 2020, 77, 105461.

[12] T. Y. Huang , C. H. Chen , C. C. Lin , Y. J. Lee , C. L. Liu , G. S. Liou , J. Mater. Chem. C 2019, 7, 11014.

[13] C. H. Chen , Y. Wang , H. Tatsumi , T. Michinobu , S. W. Chang , Y. C. Chiu , G. S. Liou , Adv. Funct. Mater. 2019, 29, 1902991.

[14] Y. Wang , L. Yin , W. Huang , Y. Li , S. Huang , Y. Zhu , D. Yang , X. Pi , Adv. Intell. Syst. 2021, 3, 2000099.

[15] J. Yin , F. Zeng , Q. Wan , F. Li , Y. Sun , Y. Hu , J. Liu , G. Li , F. Pan , Adv. Funct. Mater. 2018, 28, 1706927.

[16] S. Dai , Y. Zhao , Y. Wang , J. Zhang , L. Fang , S. Jin , Y. Shao , J. Huang , Adv. Funct. Mater. 2019, 29, 1903700.

[17] Y. Wang , Z. Lv , J. Chen , Z. Wang , Y. Zhou , L. Zhou , X. Chen , S. T. Han , Adv. Mater. 2018, 30, 1802883. 10.1002/adma.201802883 30063261

[18] Y. Park , J. S. Lee , ACS Nano 2017, 11, 8962. 28837313 10.1021/acsnano.7b03347

[19] M. Prezioso , F. Merrikh‐Bayat , B. D. Hoskins , G. C. Adam , K. K. Likharev , D. B. Strukov , Nature 2015, 521, 61. 25951284 10.1038/nature14441

[20] K. Kim , C. L. Chen , Q. Truong , A. M. Shen , Y. A. Chen , Adv. Mater. 2013, 25, 1693. 23281020 10.1002/adma.201203116

[21] M. L. Schneider , C. A. Donnelly , S. E. Russek , B. Baek , M. R. Pufall , P. F. Hopkins , P. D. Rippard , S. P. Dresselhaus , W. H. Benz , Sci. Adv. 2018, 4, e1701329. 29387787 10.1126/sciadv.1701329 PMC5786439

[22] J. Shi , S. D. Ha , Y. Zhou , F. Schoofs , S. Ramanathan , Nat. Commun. 2013, 4, 2676. 24177330 10.1038/ncomms3676

[23] M. Kumar , S. Abbas , J. Kim , ACS Appl. Mater. Interfaces 2018, 10, 34370. 30207159 10.1021/acsami.8b10870

[24] X. Zhu , W. D. Lu , ACS Nano 2018, 12, 1242. 29357245 10.1021/acsnano.7b07317

[25] S. Dai , X. Wu , D. Liu , Y. Chu , K. Wang , B. Yang , J. Huang , ACS Appl. Mater. Interfaces 2018, 10, 21472. 29877073 10.1021/acsami.8b05036

[26] J. T. Wang , S. Takashima , H. C. Wu , Y. C. Chiu , Y. Chen , T. Isono , T. Kakuchi , T. Satoh , W. C. Chen , Adv. Funct. Mater. 2016, 26, 2695.

[27] P. Kohn , S. Huettner , H. Komber , V. Senkovskyy , R. Tkachov , A. Kiriy , R. H. Friend , U. Steiner , W. T. Huck , J. U. Sommer , M. J. Sommer , J. Am. Chem. Soc. 2012, 134, 4790. 22329563 10.1021/ja210871j

[28] J. T. Wang , S. Takshima , H. C. Wu , C. C. Shih , T. Isono , T. Kakuchi , T. Satoh , W. C. Chen , Macromolecules 2017, 50, 1442.

[29] H. Im , S. Kim , Adv. Electron. Mater. 2021, 7, 2100003.

[30] J. Kim , S. M. Kwon , C. Jo , J. S. Heo , W. B. Kim , H. S. Jung , Y. H. Kim , M. G. Kim , S. K. Park , ACS Appl. Mater. Interfaces 2020, 12, 16620. 32180407 10.1021/acsami.0c01176

[31] D. H. Lim , M. Kang , S. Y. Jang , K. Hwang , I. B. Kim , E. Jung , Y. R. Jo , Y. J. Kim , J. Kim , H. Choi , T. W. Kim , S. Mathur , B. J. Kim , D. Y. Kim , ACS Appl. Mater. Interfaces 2020, 12, 25066. 32297509 10.1021/acsami.0c02229

[32] J. F. Lin , W. C. Yen , C. Y. Chang , Y. F. Chen , W. F. Su , J. Mater. Chem. 2013, 1, 665.

[33] J. Rivnay , S. C. Mannsfeld , C. E. Miller , A. Salleo , M. F. Toney , Chem. Rev. 2012, 112, 5488. 22877516 10.1021/cr3001109

[34] Y. Diao , Y. Zhou , T. Kurosawa , L. Shaw , C. Wang , S. Park , Y. Guo , J. A. Reinspach , K. Gu , X. Gu , B. C. K. Tee , C. Pang , H. Yan , D. Zhao , M. F. Toney , S. C. B. Mannsfeld , Z. Bao , Nat. Commun. 2015, 6, 7955. 26264528 10.1038/ncomms8955 PMC4557117

[35] J. Y. Lee , C. J. Lin , C. T. Lo , J. C. Tsai , W. C. Chen , Macromolecules 2013, 46, 3005.

[36] H. K. Seo , H. Kim , J. Lee , M. H. Park , S. H. Jeong , Y. H. Kim , S. J. Kwon , T. H. Han , S. Yoo , T. W. Lee , Adv. Mater. 2017, 29, 1605587. 10.1002/adma.201605587 28117521

[37] Z. Fang , W. Chen , Y. Shi , J. Zhao , S. Chu , J. Zhang , Z. Xiao , Adv. Funct. Mater. 2020, 30, 1909754.

[38] C. Fei , H. Wang , Org. Electron. 2019, 68, 143.

[39] M. Li , B. Li , G. Cao , J. Tian , J. Mater. Chem. A 2017, 5, 21313.

[40] X. Yu , K. Xiao , J. Chen , N. V. Lavrik , K. Hong , B. G. Sumpter , D. B. Geohegan , ACS Nano 2011, 5, 3559. 21456581 10.1021/nn2007964

[41] J. Zhang , S. Dai , Y. Zhao , J. Zhang , J. Huang , Adv. Intell. Syst. 2020, 2, 1900136.

[42] H. T. Hsu , D. L. Yang , L. D. Wiyanto , J. Y. Chen , Adv. Photonics Res. 2021, 2, 20000185.

[43] T. B. Singh , R. Koeppe , N. S. Sariciftci , M. Morana , C. J. Brabec , Adv. Funct. Mater. 2009, 19, 789.

[44] C. T. Herrera , M. J. Hong , J. G. Labram , ACS Appl. Electron. Mater. 2020, 2, 2257.

[45] L. Shao , Y. Zhao , Y. Liu , Adv. Funct. Mater. 2021, 31, 2101951.

[46] Y. Li , Y. Wang , L. Yin , W. Huang , W. Peng , Y. Zhu , K. Wang , D. Yang , X. Pi , Sci. China Inf. Sci. 2021, 64, 162401.

[47] L. Yin , X. Pi , D. Yang , Chin. Phys. B 2020, 29, 070703.

[48] L. Yin , C. Han , Q. Zhang , Z. Ni , S. Zhao , K. Wang , D. Li , M. Xu , H. Wu , X. Pi , D. Yang , Nano Energy 2019, 63, 103859.

[49] K. Wang , S. Dai , Y. Zhao , Y. Wang , C. Liu , J. Huang , Small 2019, 15, 1900010. 10.1002/smll.201900010 30740892

[50] Q. Ou , B. Yang , J. Zhang , D. Liu , T. Chen , X. Wang , D. Hao , Y. Lu , J. Huang , Small 2021, 17, 2007241. 10.1002/smll.202007241 33590701

[51] B. Yang , Y. Lu , D. Jiang , Z. Li , Y. Zeng , S. Zhang , Y. Ye , Z. Liu , Q. Ou , Y. Wang , S. Dai , Y. Yi , J. Huang , Adv. Mater. 2020, 32, 2001227. 10.1002/adma.202001227 32500583

[52] D. Hao , J. Zhang , S. Dai , J. Zhang , J. Huang , ACS Appl. Mater. Interfaces 2020, 12, 39487. 32805934 10.1021/acsami.0c10851

[53] E. Ercan , Y.‐C. Lin , W.‐C. Yang , W.‐C. Chen , Adv. Funct. Mater. 2021, 31, 2107925.

[54] J.‐Y. Chen , D.‐L. Yang , F.‐C. Jhuang , Y.‐H. Fang , J.‐S. Benas , F.‐C. Liang , C.‐C. Kuo , Adv. Funct. Mater. 2021, 31, 2105911.

[55] Y. Zhai , J.‐Q. Yang , Y. Zhou , J.‐Y. Mao , Y. Ren , V. A. L. Roy , S.‐T. Han , Mater. Horiz. 2018, 5, 641.

[56] Y.‐C. Lin , W.‐C. Yang , Y.‐C. Chiang , W.‐C. Chen , Small Sci. 2021, 1, 2100109. 10.1002/smsc.202100109 PMC11935926 40212670

[57] N. G. Kang , B. Cho , B. G. Kang , S. Song , T. Lee , J. S. Lee , Adv. Mater. 2012, 24, 385. 22162078 10.1002/adma.201103862

[58] K. J. Baeg , Y. Y. Noh , J. Ghim , B. Lim , D. Y. Kim , Adv. Funct. Mater. 2008, 18, 3678.

[59] X. J. She , J. Liu , J. Y. Zhang , X. Gao , S. D. Wang , Appl. Phys. Lett. 2013, 103, 143302.

