# Supplementary material for: Low‐Energy‐Consumption and Electret‐Free Photosynaptic Transistor Utilizing Poly(3‐hexylthiophene)‐Based Conjugated Block Copolymers
Source: Adv Sci (Weinh). 2022 Jan 22;9(8):2105190. doi: 10.1002/advs.202105190 (PMC8922097; doi:10.1002/advs.202105190)
Supplement: Supplementary file 1 — Supporting Information [file ADVS-9-2105190-s001.pdf]

ADVANCED SCIENCE
Open Access

Supporting Information

for *Adv. Sci.*, DOI 10.1002/advs.202105190

Low-Energy-Consumption and Electret-Free Photosynaptic Transistor Utilizing Poly(3-hexylthiophene)-Based Conjugated Block Copolymers

Wei-Chen Yang, Yan-Cheng Lin, Shin Inagaki, Hiroya Shimizu, Ender Ercan, Li-Che Hsu, Chu-Chen Chueh, Tomoya Higashihara and Wen-Chang Chen**

ADVANCED SCIENCE
Open Access

Supporting Information

for *Adv. Sci.*, DOI: 10.1002/advs.202105190

Low-Energy-Consumption and Electret-Free Photosynaptic Transistor Utilizing Poly(3-hexylthiophene)-Based Conjugated Block Copolymers

Wei-Chen Yang,[a,b] *Yan-Cheng Lin,*[a,b] *Shin Inagaki,*[c] *Hiroya Shimizu,*[c] *Ender Ercan,*[a.b] *Li-Che Hsu,*[b,d] *Chu-Chen Chueh,*[a,b] *Tomoya Higashihara,*[c*] *and Wen-Chang Chen,*[a,b*]

Supporting Information

Low-Energy-Consumption and Electret-Free Photosynaptic Transistor Utilizing Poly(3-hexylthiophene)-Based Conjugated Block Copolymers

Wei-Chen Yang,[a,b] *Yan-Cheng Lin,*[a,b] *Shin Inagaki,*[c] *Hiroya Shimizu,*[c] *Ender Ercan,*[a.b] *Li-Che Hsu,*[b,d] *Chu-Chen Chueh,*[a,b] *Tomoya Higashihara,*[c*] *and Wen-Chang Chen,*[a,b*]

[a] W.-C. Yang, Dr. Y.-C. Lin, Dr. E. Ercan, Prof. C.-C. Chueh and Prof. W.-C. Chen
Department of Chemical Engineering, National Taiwan University, Taipei 10617, Taiwan

[b] W.-C. Yang, Dr. Y.-C. Lin, Dr. E. Ercan, Dr. L.-C. Hsu, Prof. C.-C. Chueh and Prof. W.-C. Chen
Advanced Research Center for Green Materials Science and Technology, National Taiwan University, Taipei 10617, Taiwan

[c] S. Inagaki, H. Shimizu, Prof. T. Higashihara
Department of Organic Materials Science, Graduate School of Organic Materials Science, Yamagata University, Yonezawa, Yamagata 992-8510

[d] Dr. L.-C. Hsu
Institute of Polymer Science and Engineering, National Taiwan University, Taipei 10617, Taiwan

*Corresponding author. E-mail:
chenwc@ntu.edu.tw (W.-C. Chen); thigashihara@yz.yamagata-u.ac.jp (T. Higashihara)

Synthesis of polymers.

As shown in **Scheme S1**, the synthesis of poly(3-hexylthiophene)-*b*-poly(2-vinylpyridine) (P3HT-*b*-P2VP) and poly(3-hexylthiophene)-*b*-poly(2-vinylnaphthalene) (P3HT-*b*-PVN) were synthesized by the copper-catalyzed azide-alkyne cycloaddition (CuAAC) reaction between chain-end-functionalized polymers with an alkynyl or azide group which were prepared according to the previous reports. [1-4]

Synthesis of P3HT-*b*-P2VP.

0.11 g (0.014 mmol) of ω-chain-end-functionalized P3HT with an alkynyl group (P3HT-Alkyne; $M_{n,NMR}$ = 8000, $M_{n,SEC}$ = 14000 (calibrated using polystyrene standards), $Đ_{M,SEC}$ = 1.05),[1] 0.24 g (0.034 mmol) of α-chain-end-functionalized P2VP (N_3-P2VP; $M_{n,NMR}$ = 7100, $M_{n,SEC}$ = 6200 (calibrated using polystyrene standards), $Đ_{M,SEC}$ = 1.14),[2] and 0.053 g (0.31 mmol) of *N,N,N′,N″,N″*-pentamethyldiethylenetriamine (PMDETA) were placed in a flask under a nitrogen atmosphere and dissolved in 5.0 mL of tetrahydrofuran (THF). After the freeze-pump-thaw cycling for three times, copper bromide (CuBr; 0.043 g, 0.30 mmol) was added to the solution and refluxed overnight. The resulted polymer was then purified by Soxhlet extraction using acetone and hexane under nitrogen, and recovered with chloroform. Note that the Soxhlet extraction of the polymer at elevated temperature seems to lead to the somewhat lower M_n value than the theoretical one; therefore, the reprecipitation using good and poor solvents may be better way for the workup. The polymer was finally freeze-dried from its absolute benzene solution to afford P3HT-*b*-P2VP (with P3HT homopolymer contamination of ca. 37 mol%). $M_{n,NMR}$ = 11000, $M_{n,SEC}$ = 13000 (calibrated using polystyrene standards), $Đ_{M,SEC}$ = 1.14, P3HT:P2VP = 64:36, *w*:*w* (determined by ^{1}H NMR),

120 mg, 58%.

Synthesis of α-chain-end-functionalized PVN with an alkynyl group (Alkyne-PVN).

1.0 g (6.5 mmol) of 2-vinylnaphthalene, 0.025 g (0.064 mmol) of 2-(dodecylsulfanylthiocarbonyl)sulfanyl)propanoic acid propargyl ester,[3] and 0.003 g (0.018 mmol) of 2,2'-azobis(isobutyronitrile) (AIBN) were placed in a flask and dissolved in 3.3 mL of toluene. After bubbling of the solution with nitrogen, it was stirred at 65 °C overnight under nitrogen to proceed the polymerization. The solution was cooled down in a dewar of liquid nitrogen and powered into hexane to precipitate the polymer. It was freeze-dried from its absolute benzene solution to afford Alkyne-PVN. $M_{n,SEC}$ = 5200 (calibrated using polystyrene standards), $Đ_{M,SEC}$ = 1.22, 317 mg, 32%.

Synthesis of P3HT-*b*-PVN.

0.10 g (0.018 mmol) of α-chain-end-functionalized P3HT with an azide group (N_3-P3HT; $M_{n,NMR}$ = 5500, $M_{n,SEC}$ = 8100 (calibrated using polystyrene standards), $Đ_{M,SEC}$ = 1.10),[4] 0.15 g (0.029 mmol) of Alkyne-PVN ($M_{n,SEC}$ = 5200 (calibrated using polystyrene standards), $Đ_{M,SEC}$ = 1.22), and 0.043 g (0.25 mmol) of PMDETA were placed in a flask under a nitrogen atmosphere and dissolved in 5.0 mL of THF. After the freeze-pump-thaw cycling for three times, CuBr (0.036 g, 0.25 mmol) was added to the solution and at 60 °C or 1.5 h. The solution was poured into methanol to precipitate the polymer. It was then purified by Soxhlet extraction using methanol, acetone, and hexane under nitrogen, and recovered with chloroform. The polymer was finally dried by freeze-drying from its absolute benzene solution to afford P3HT-*b*-PVN. $M_{n,NMR}$ = 12000, $M_{n,SEC}$ =

15000 (calibrated using polystyrene standards), $Đ_{M,SEC}$ = 1.15, P3HT:PVN = 46:54, *w*:*w* (determined by ^{1}H NMR), 150 mg, 77%.

Characterization.

^{1}H nuclear magnetic resonance (^{1}H NMR) spectra were recorded on a JEOL JNM-ECX400 (400 MHz) in deuterated chloroform-d_1 ($CDCl_3$) or 1,1,2,2-tetrachloroethane-d_2 ($C_2D_2Cl_4$) at 25 °C. The M_n and $Đ_M$ values were measured by size exclusion chromatography (SEC) using a JASCO GULLIVER HPLC system equipped with a pump (JASCO PU-1580), a column oven (JASCO CO-2065Plus), a UV detector (λ = 254 nm, JASCO UV-1575), and an RI detector (RI-1580). The column set was as follows: a guard column (Shodex KF-G) and two consecutive columns (Shodex KF-804L and Shodex KF-805L) eluted with THF at 40 °C at a flow rate of 1.0 mL/min. Polystyrene standards were used for the calibration. Thermogravimetric analysis (TGA) was conducted by using a Discovery TGA 25 (TA Instruments). 3-5 mg powder samples were heated under the nitrogen flow at a heating rate of 10 °C/min from 100 to 700 °C. Differential scanning calorimetry (DSC) analysis was performed on Discovery DSC 25 (TA Instruments), in which 3-5 mg powder samples at a heating rate of 10 °C/min from -80 to 250 °C was tested. The film thickness of P3HT-based BCPs were measured by using a surface profilometer, Surfcorder ET3000. Atomic force microscopy (AFM) images of the polymer films were obtained by using Nanoscope 3D controller AFM (Digital Instruments) operated in the tapping mode. UV-vis absorption spectra were recorded using a U-4100, Hitachi spectrometer and the photoluminescence (PL) emissions were measured using a Horiba Fluorolog-3 spectrometer system. The time-resolved

photoluminescence (TRPL) spectra of the polymer films were excited at wavelength of 405 nm and conducted in Photonic Workshop (Center for Condensed Matter Sciences, National Taiwan University). Cyclic voltammetry (CV) was performed by a CHI 6273E electrochemical analyzer using a three-electrode cell system, where an ITO glass was used as the working electrode and a platinum wire was used as the auxiliary electrode. Ag/AgCl, KCl (sat.) was used as the reference electrode. The electrochemical properties of the polymer films were measured in 0.1 M dry acetonitrile solution containing tetra-*n*-butylammonium perchlorate as the electrolyte. The onset oxidation potentials (E_{onset}^{ox}) for the BCPs and the reference redox couple of ferrocene ($E_{ferrocene}^{1/2}$) were used to determine the HOMO levels according to the equation HOMO (eV) = $-e[E_{onset}^{ox} - E_{ferrocene}^{1/2} + 4.8]$. The lowest unoccupied molecular orbital (LUMO) levels were calculated by using the HOMO levels and the optical bandgaps. The solid-state stacking and crystallographic properties of the polymer films were characterized by grazing incidence wide angle X-ray diffraction (GIXD) on beamline 13A1 in National Synchrotron Radiation Research Center (NSRRC), Taiwan with monochromatic beams wavelength of 1.0273 Å. and an incident angle of 0.12^{o}.

Fabrication the space charge limited current (SCLC) and capacitance devices.

The hole-only device were fabricated with the structure of ITO/ MoO_3/ P3HT-based BCPs/ MoO_3/ Al. The space charge limited current (SCLC) measurement was employed by using a Keithley 4200-SCS semiconductor parameter analyzer (Tektronix) in a nitrogen-filled glovebox in the dark environment under bias from 0 V to 7 V. The capacitances of P3HT-based BCPs were

determined by using a metal-oxide-semiconductor (MOS) architecture of Si/ SiO_2/ P3HT-based BCPs/ Au configuration and characterized through a Keithley 4200-SCS semiconductor parameter analyzer (Tektronix) in a nitrogen-filled glovebox.

Table S1. Optical and electrochemical properties of the BCPs studied and their constituent homopolymers.

Sample	λ_{max} (nm)	E_g^{opt} (eV)	HOMO (eV)	LUMO (eV)
P3HT-*b*-PS	521, 558, 601	1.90	-5.29	-3.39
P3HT-*b*-P2VP	264, 531, 555, 602	1.83	-5.26	-3.43
P3HT-*b*-PVN	232, 280, 531, 595	1.88	-5.29	-3.41
P3HT-*b*-PBA	523, 553, 602	1.85	-5.28	-3.43
P3HT	272, 531, 595	2.00	-5.21	-3.21
PS	258	4.56	-6.22	-1.66
P2VP	263	4.46	-6.15	-1.69
PVN	230, 282	3.79	-5.61	-1.82
PBA	229	4.80	-6.53	-1.73

Table S2. TR-PL fitting parameters of the BCP films.

Sample	τ_1 (ns)	A_1	τ_2 (ns)	A_2	τ_{avg} (ns)
**P3HT-*b*-PS**	0.113 ± 0.004	227 ± 9	0.828 ± 0.052	10.0 ± 1.0	0.29 ± 0.03
**P3HT-*b*-P2VP**	$(59.7 \pm 1.4)\times10^{-3}$	30200 ± 800	0.335 ± 0.015	1640 ± 110	0.23 ± 0.02
**P3HT-*b*-PVN**	$(3.54 \pm 0.1)\times10^{-3}$	23200 ± 1300	0.199 ± 0.006	421 ± 15	0.24 ± 0.02
**P3HT-*b*-PBA**	$(14.3 \pm 0.3)\times10^{-3}$	50900 ± 1900	0.225 ± 0.033	269 ± 57	0.09 ± 0.02

The carrier lifetime (τ) is fitted by using a reconvolution method. The data processing using reconvolution techniques does not aim a retrieving the raw data as seen by an ideal instrument, but usually compares decays (simulated on the base of a model) that are convolved with the measured IRF by numerically solving the convolution integral. Therefore, we utilized the intensity weighted method to calculate the τ_{avg}, as the follow equation: $\tau_{avg} = \frac{\sum_{i=1}^{n} A_i\tau_i^2}{\sum_{i=1}^{n} A_i\tau_i}$

Table S3. The dielectric constant (D_k), trap-filled limit voltage (V_{TFL}), trap density (N_{trap}), and SCLC mobility (μ_{SCLC}) of the BCP films.

Sample	P3HT-*b*-PS	P3HT-*b*-P2VP	P3HT-*b*-PVN	P3HT-*b*-PBA
D_k	3.0	4.0	4.3	4.0
V_{TFL} (V)	2.9	3.4	4.3	3.1
N_{trap} (m^{-3})	6.65×10^{24}	4.91×10^{24}	7.39×10^{24}	5.52×10^{25}
μ_{SCLC} ($cm^2V^{-1}s^{-1}$)	4×10^{-9}	1.29×10^{-8}	1.28×10^{-10}	5.42×10^{-10}

Table S4. Memory parameters of the phototransistors with thermally annealed BCP films as a channel.

Sample	λ(nm)	μ_h (cm^2 V^{-1} s^{-1})	I_{ON}/I_{OFF}	ΔV_{th} (V)
P3HT-*b*-PS	254 nm	2.198×10^{-3}	10^3	28.1
	405 nm	2.465×10^{-3}	10^4	29.9
	530 nm	3.116×10^{-3}	10^4	26.3
	650 nm	3.021×10^{-3}	10^4	26.1
P3HT-*b*-P2VP	254 nm	1.662×10^{-3}	10^5	38.3
	405 nm	1.660×10^{-3}	10^5	31.2
	530 nm	1.668×10^{-3}	10^5	29.0
	650 nm	1.789×10^{-3}	10^4	26.7
P3HT-*b*-PVN	254 nm	4.355×10^{-5}	10^2	3.7
	405 nm	3.284×10^{-5}	10^1	1.6
	530 nm	4.179×10^{-5}	10^2	3.2
	650 nm	3.472×10^{-5}	10^1	3.2
P3HT-*b*-PBA	254 nm	9.316×10^{-5}	10^2	24.2
	405 nm	8.307×10^{-5}	10^3	25.6
	530 nm	8.613×10^{-5}	10^3	28.5
	650 nm	8.936×10^{-5}	10^3	24.7

Table S5. PPF fitting parameters of the synaptic transistors with the BCP films as channels.

		**P3HT-*b*-PS**		**P3HT-*b*-P2VP**		**P3HT-*b*-PBA**	
		τ_1 (ms)	τ_2 (ms)	τ_1 (ms)	τ_2 (ms)	τ_1 (ms)	τ_2 (ms)
Light wavelength (nm; 22 mW/cm^2)	450	0.006	0.570	0.033	593	0.012	0.37
	530	--	--	0.005	0.72	--	--
	650	--	--	0.006	0.67	--	--
Light intensity (mW/cm^2; 450 nm)	2.2	--	--	0.011	27	--	--
	18	--	--	0.012	547	--	--
	22	0.006	0.570	0.033	593	0.012	0.37

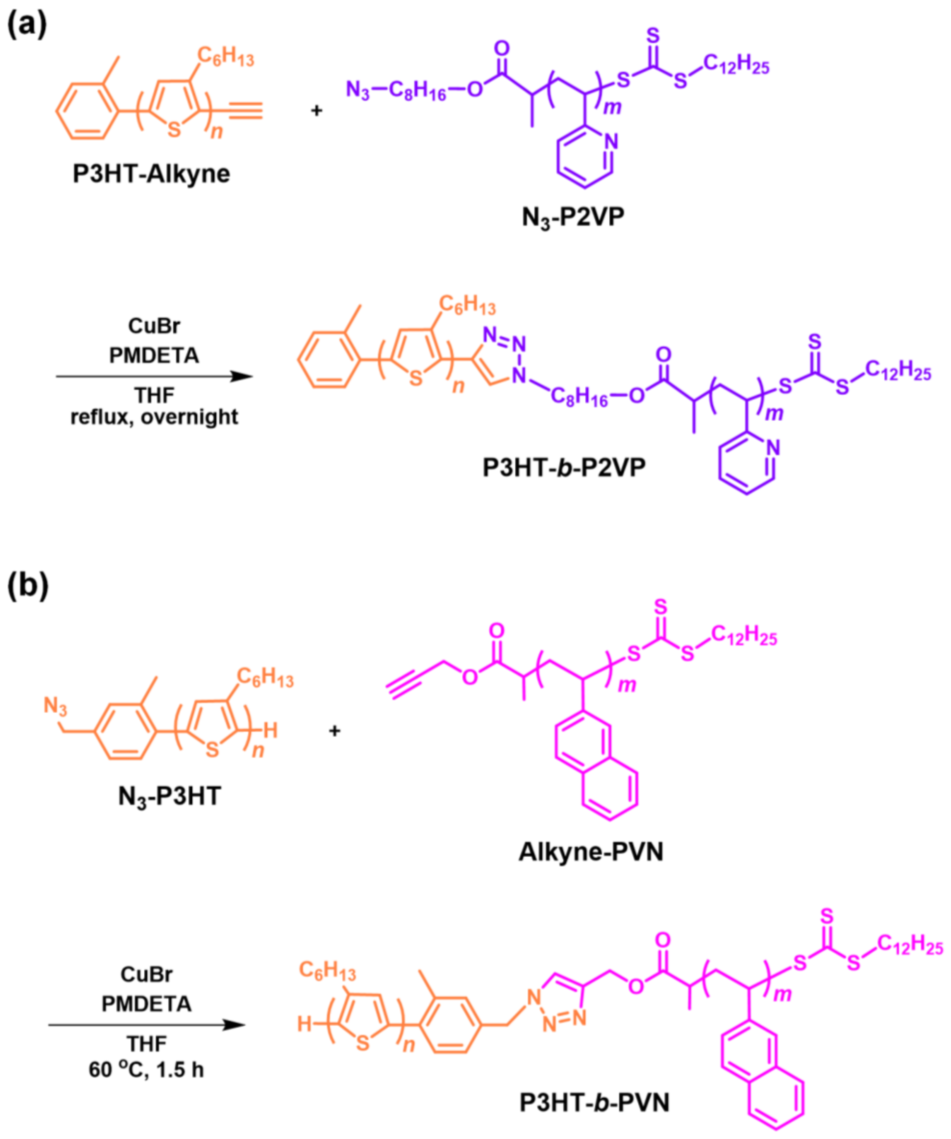

Scheme S1. Synthetic routes for (a) P3HT-*b*-P2VP and (b) P3HT-*b*-PVN.

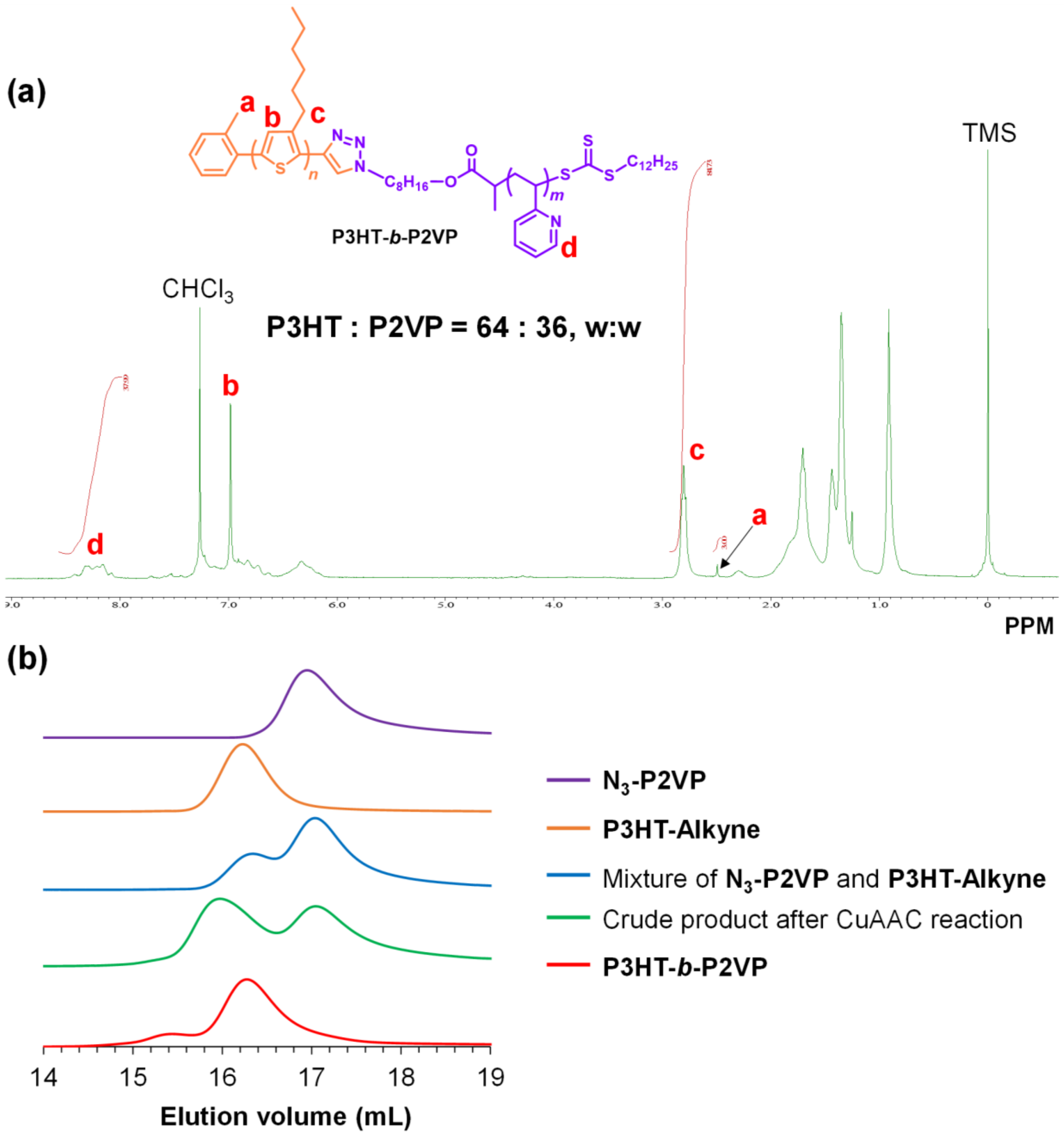

Figure S1. (a) ^{1}H NMR spectrum of P3HT-*b*-P2VP in $CDCl_3$. (b) SEC profiles of N_3-P2VP, P3HT-Alkyne, the mixture of N_3-P2VP and P3HT-Alkyne before the CuAAC reaction between N_3-P2VP and P3HT-Alkyne, crude product obtained after the CuAAC reaction between N_3-P2VP and P3HT-Alkyne, and P3HT-*b*-P2VP with an eluent of THF at a rate of 1.0 mL/min.

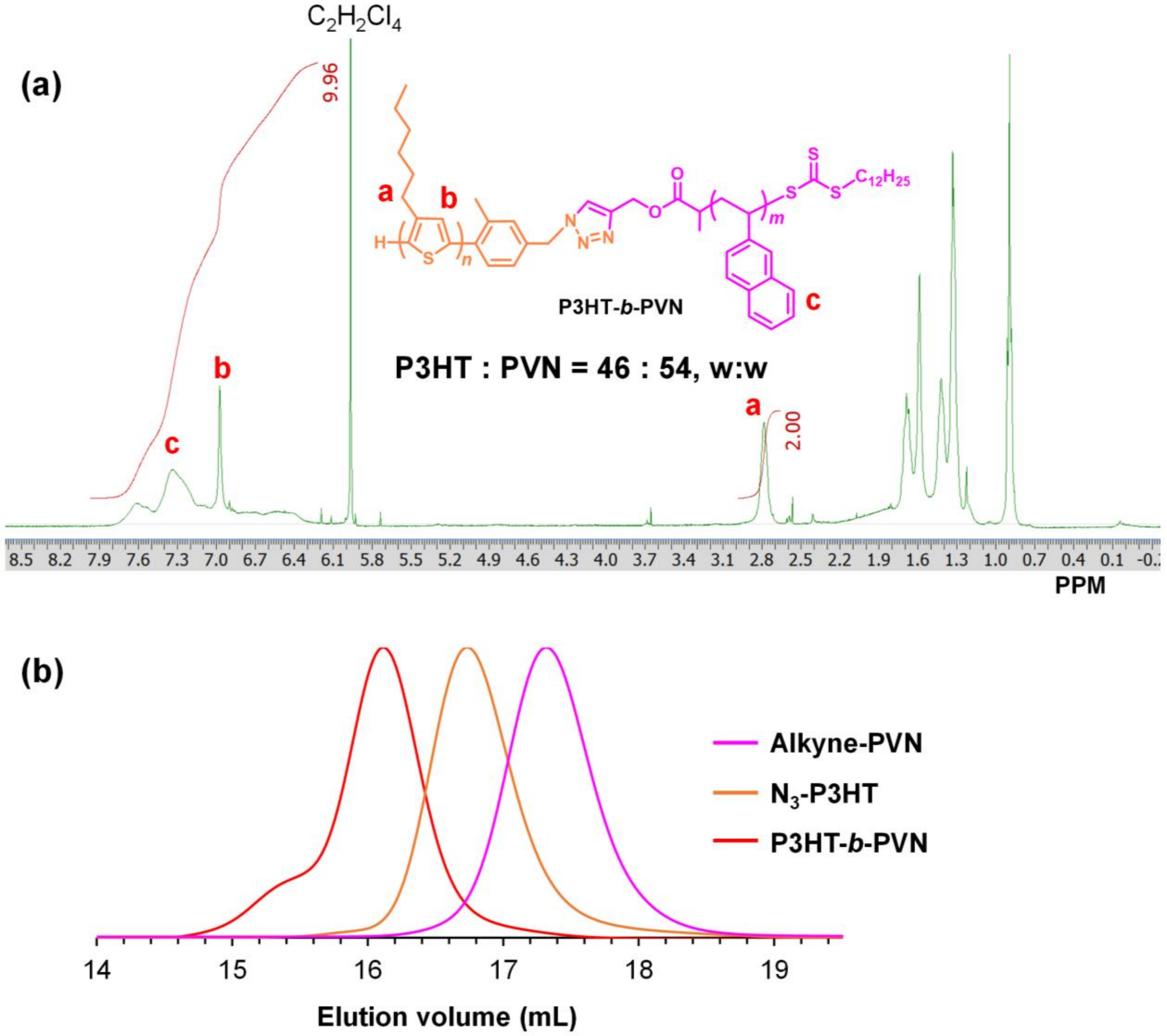

Figure S2. (a) ^{1}H NMR spectrum of P3HT-*b*-PVN in $C_2D_2Cl_4$. (b) SEC profiles of Alkyne-PVN, N_3-P3HT, and P3HT-*b*-PVN with an eluent of THF at a rate of 1.0 mL/min.

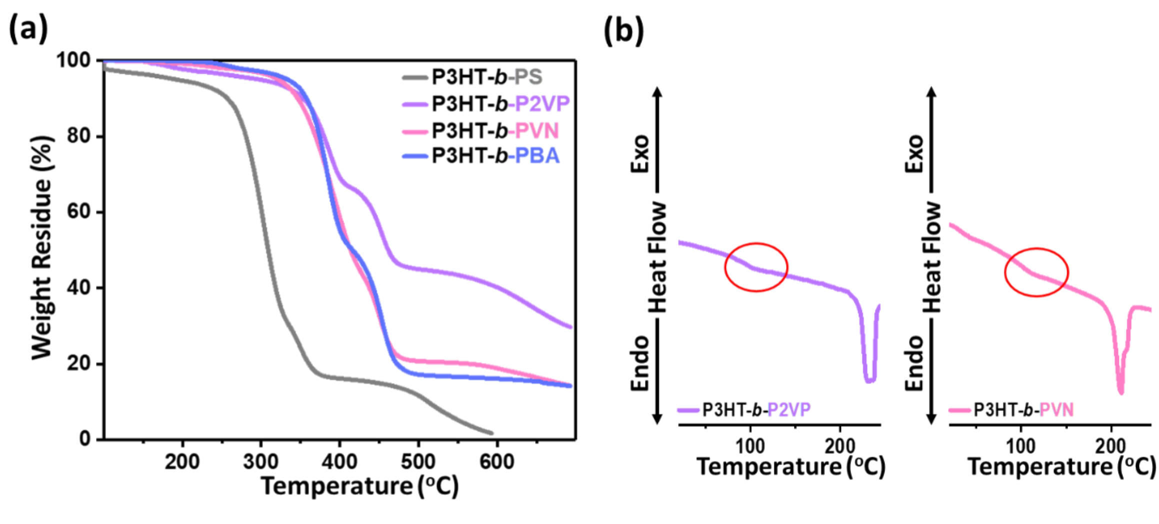

Figure S3. (a) TGA traces of the P3HT-based BCPs. (b) Enlarge DSC traces of the second heating process of P3HT-*b*-P2VP and P3HT-*b*-PVN.

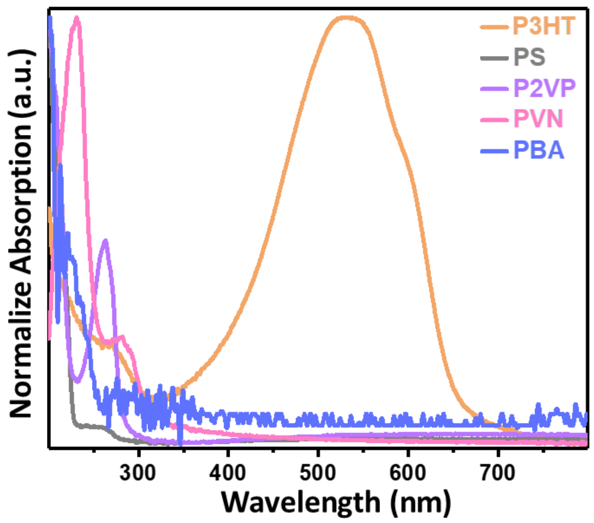

Figure S4. UV-vis absorption spectra of the constituent homopolymers.

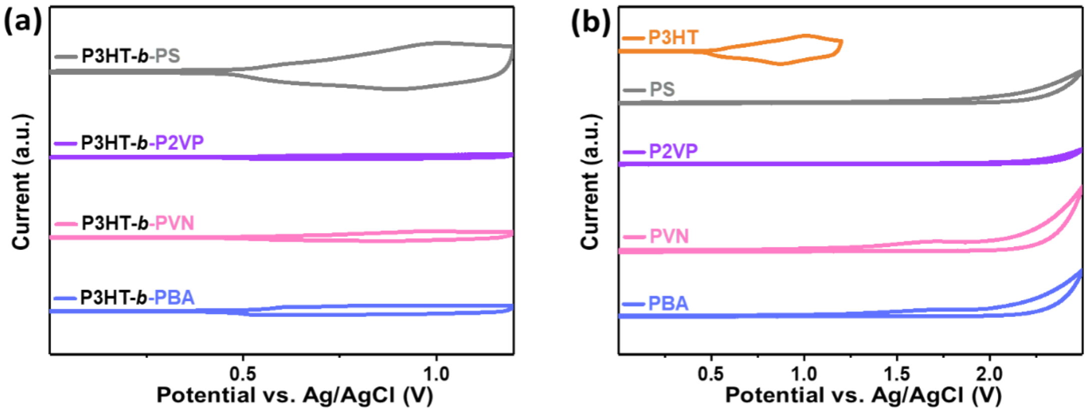

Figure S5. CV profiles of (a) the BCP films and (b) constituent homopolymer films.

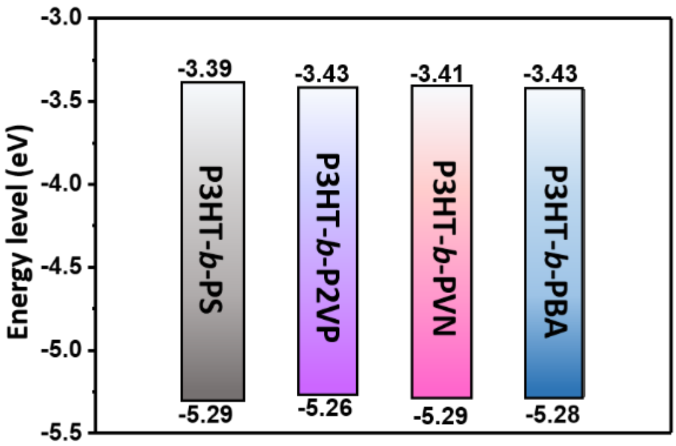

Figure S6. Energy level diagram of the P3HT-based BCP films.

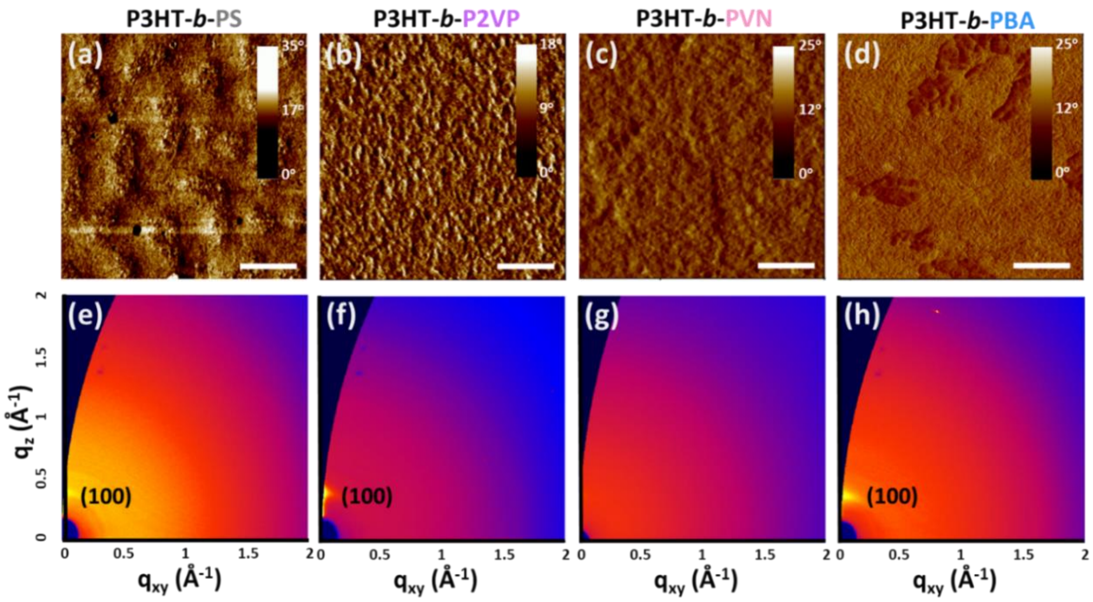

Figure S7. (a-d) AFM phase images with inset scale bar of 2.8 μm and (e-h) 2D GIXD profiles of the as-cast BCP films of (a,e) P3HT-*b*-PS, (b,f) P3HT-*b*-P2VP, (c,g) P3HT-*b*-PVN, and (d,h) P3HT-*b*-PBA.

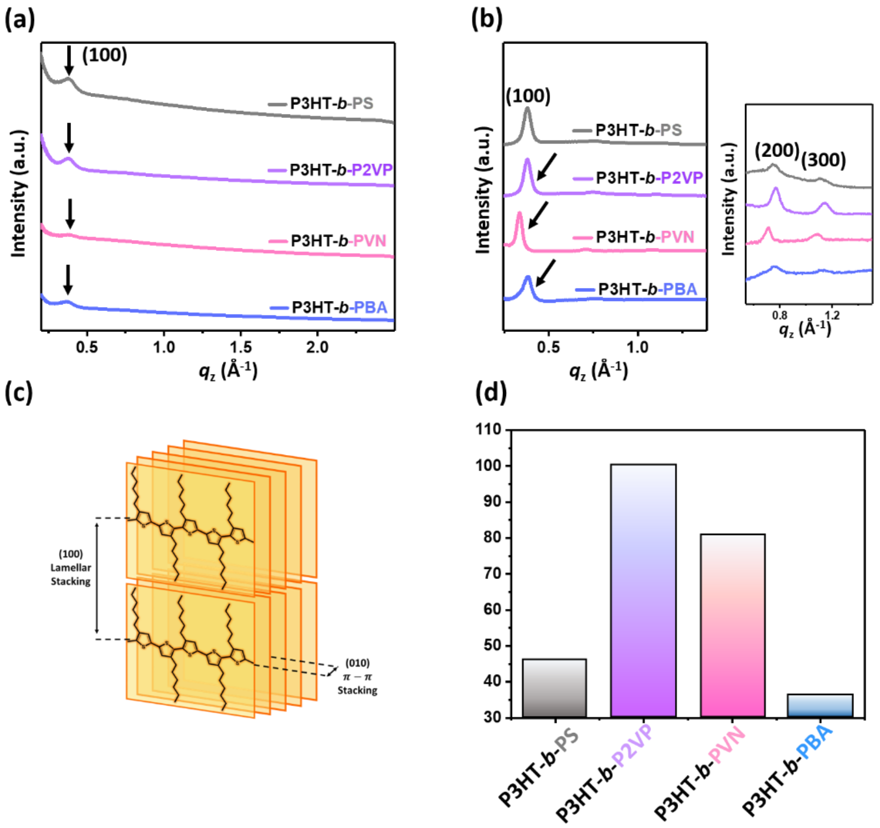

Figure S8. 1D GIXD profiles in the out-of plane direction of the (a) as-cast and (b) thermally annealed BCP films. (c) Schematic illustration of the stacking patterns of the P3HT domain. (d) The relative degree of crystallinity of the thermally annealed BCP films.

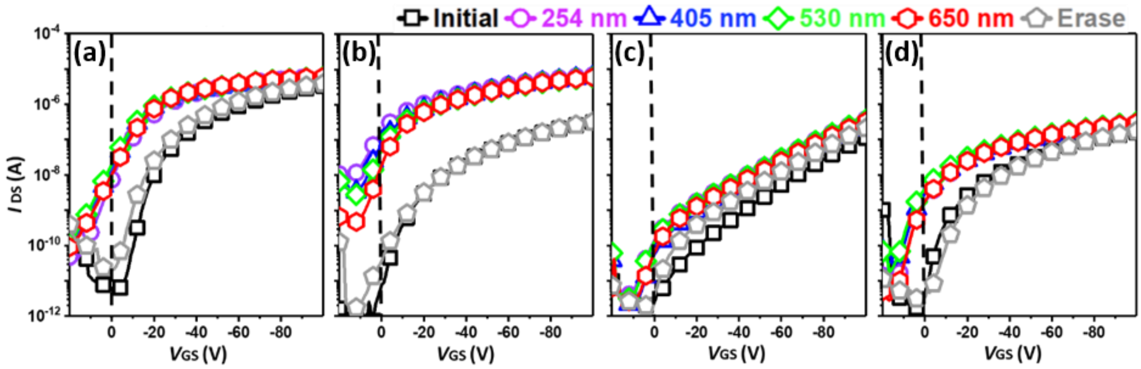

Figure S9. Transfer curves of the phototransistors with thermally-annealed BCP films of (a) P3HT-*b*-PS, (b) P3HT-*b*-P2VP, (c) P3HT-*b*-PVN, and (d) P3HT-*b*-PBA with photo-writing of 254 nm, 0.916 mW/cm^2; 405 nm, 10 mW/cm^2; 530, 10 mW/cm^2; 650 nm, 8 mW/cm^2 for 40 s and electrical erasing with V_{GS} of −100 V. The transfer curves were collected at V_{DS} of −100 V.

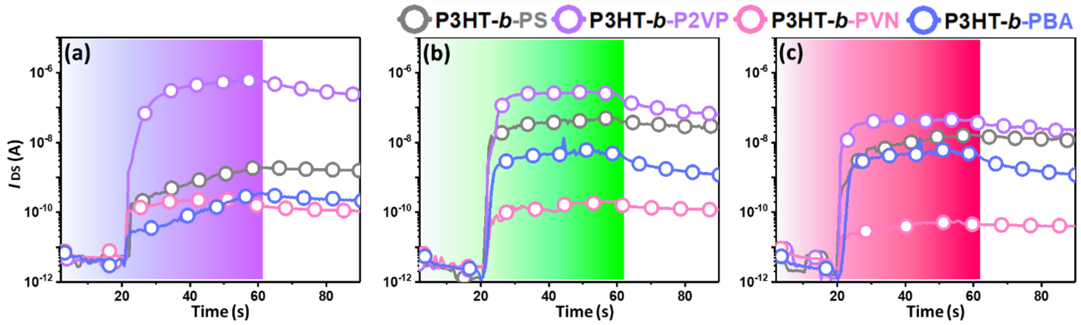

Figure S10. Temporal I_{DS} curves of the phototransistors comprising the thermally annealed BCP films with photo-writing of (a) 254 nm, 0.916 mW/cm^2; (b) 530, 10 mW/cm^2; (c) 650 nm, 8 mW/cm^2 for 40 s at V_{DS} of −100 V. The color regions represent the light illumination.

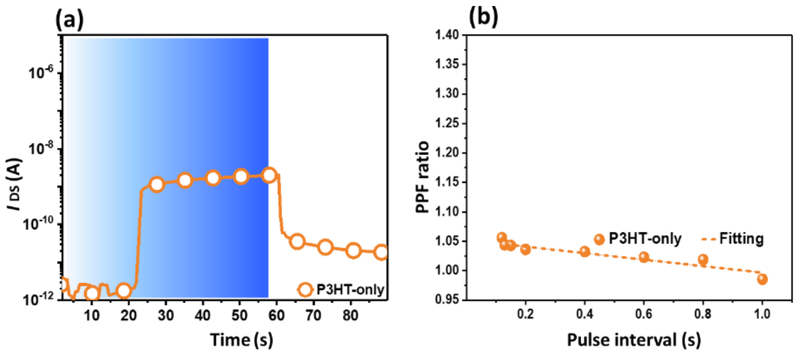

Figure S11. (a) Temporal I_{DS} curves of the phototransistor memory comprising P3HT as a channel. The color regions in the temporal I_{DS} curves represent the light illumination with photo-writing of 405 nm, 10 mW/ cm^2 for 40 s at V_{DS} of −100V. (b) Variations of PPF to the light pulse intervals of the photosynaptic transistor comprising P3HT as a channel. The EPSC was tracked by giving the presynaptic light pulses with a width of 0.2 s, light intensity of 22 mW/cm^2, and light wavelength of 450 nm.

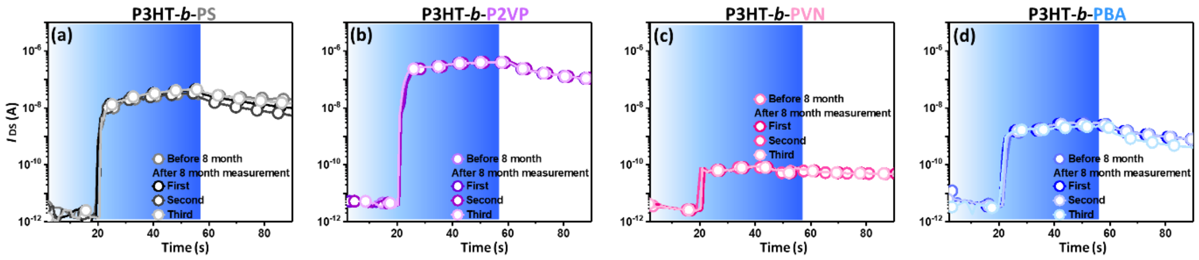

Figure S12. Comparison of the temporal I_{DS} curves of the fresh BCP films and those of stored in nitrogen atmosphere for 8 months: (a) P3HT-*b*-PS, (b) P3HT-*b*-P2VP, (c) P3HT-*b*-PVN, and (d) P3HT-*b*-PBA. The color regions in the temporal I_{DS} curves represent the light illumination with photo-writing of 405 nm, 10 mW/ cm^2 for 40 s at V_{DS} of −100V.

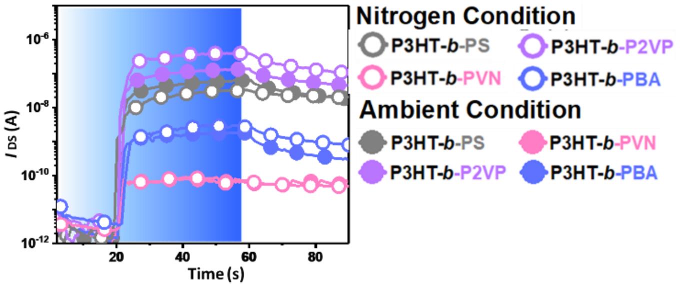

Figure S13. Comparison of the temporal I_{DS} curves of BCP films measured in nitrogen or ambient conditions with photo-writing of 405 nm, 10 mW/ cm^2 for 40 s at V_{DS} of −100V. The color regions in the temporal I_{DS} curves represent the light illumination.

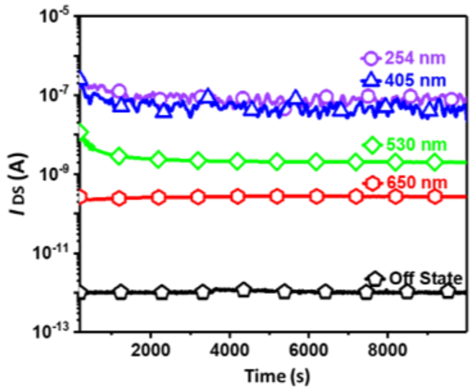

Figure S14. Long-term stability of the device with P3HT-*b*-P2VP film conducted with varied light illumination and the I_{DS} was collected at V_{DS} of −100 V.

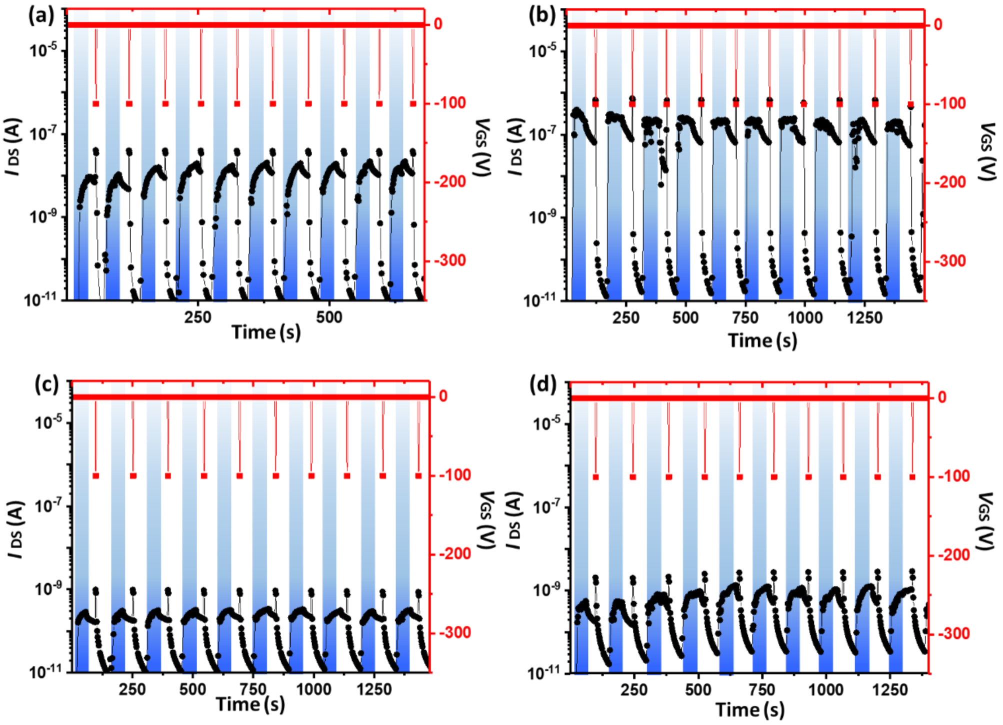

Figure S15. Memory endurance tests of the devices with the thermally annealed (a) P3HT-*b*-PS, (b) P3HT-*b*-P2VP, (c) P3HT-*b*-PVN, and (d) P3HT-*b*-PBA films conducted with photo-writing of 405 nm, 10 mW/cm^2, 40 s and electrical erasing with V_{GS} of −100 V. The I_{DS} was collected at V_{DS} of −100 V.

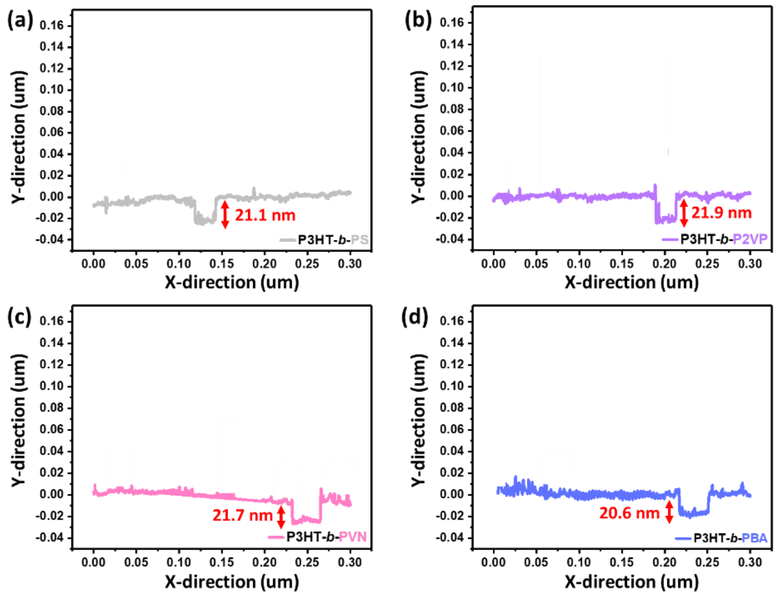

Figure S16. Surface profiles of the P3HT-based BCP films: (a) P3HT-*b*-PS, (b) P3HT-*b*-P2VP, (c) P3HT-*b*-PVN, and (d) P3HT-*b*-PBA.

References:

[1] M. Chevrier, G. Lopez, W. Zajaczkowski, J. Kesters, R. Lenaerts, M. Surin, J. De Winter, S. Richeter, W. Pisula, A. Mehdi, P. Gerbaux, R. Lazzaroni, P. Dubois, W. Maes, B. Ameduri, S. Clément, *Synth. Met.* **2019**, *252*, 127.

[2] M.T ian, C. Ma, X. Huang, G. Lu, C. Feng, *Polym. Chem.* **2021**, *12*, 1924.

[3] The synthetic protocol is as follows: In the two-neck flask, 2-(dodecylthiocarbonothioylthio)propionic acid (2.02 g, 5.8 mmol), propargyl alcohol (0.41 g, 7.3 mmol), and 4-dimethylaminopyridine (0.035 g, 0.29 mmol) were placed and dried under vacuum. The flask was then purged with nitrogen. After dissoving reactants in dichloromethane (50 mL) and cooling to 0 °C, 1-(3-dimethylaminopropyl)-3-ethylcarbodiimide (1.2 mL, 8.8 mmol) was dropwisely added to the solution. It was warmed to room temperature and stirred overnight. After washing the solution with water, the product was extracted with dichloromethane. The solution was dried over magnesium sulfate and filtered. The filtrate was concentrated under the reduced pressure and the product was dried under vacuum. The purification of the product by silicagel column chromatography (eluent: hexane:dichloromethane = 8:2, v:v) afforded 2-(dodecylsulfanylthiocarbonyl)sulfanyl)propanoic acid propargyl ester (1.06 g, 47%). ^{1}H NMR (400MHz, $CDCl_3$) δ 4.82 (q, *J* = 7.4 Hz), 4.71 (t, *J* = 2.5 Hz), 3.33 (t, *J* = 7.5 Hz), 2.48 (t, *J* = 2.5 Hz), 1.71-1.63 (m), 1.59 (d, *J* = 7.7 Hz), 1.36 (q, *J* = 6.8 Hz), 1.24 (s), 0.86 (t, *J* = 6.8Hz).

[4] M.-P. Van Den Eede, J. De Winter, P. Gerbaux, J. Teyssandier, S. De Feyter, C. Van Goethem, I. F. J.Vankelecom, G. Koeckelberghs, *Macromolecules* **2018**, *51*, 8689.
